# Association between coffee and tea consumption and the risk of macrovascular complications in type 2 diabetes: a UK Biobank cohort study

**DOI:** 10.1186/s13098-025-01807-4

**Published:** 2025-06-19

**Authors:** Ting Ma, Lingling Yang, Miaomiao Wu, Bo Wang, Jiangping Li, Jiafei Yang, Xian Sun

**Affiliations:** 1 School of Public Health, Ningxia Medical University, Yinchuan, 750004 China; 2 Key Laboratory of Environmental Factors and Chronic Disease Control, Ningxia Medical University, Yinchuan, 750004 China

**Keywords:** Coffee consumption, Tea consumption, Macrovascular complications, Type 2 diabetes mellitus

## Abstract

**Background:**

Many studies have shown that coffee and tea consumption is associated with diabetes. However, limited research exists on their effects on the risk of macrovascular complications in diabetic patients. Therefore, the purpose of this study was to examine the relationship between the intake of coffee and tea and macrovascular complications in patients with type 2 diabetes mellitus (T2DM).

**Methods:**

We used the Cox proportional hazards regression model to estimate the hazard ratio (HR) and 95% confidence interval (CI), which determined the relationship between coffee and tea consumption and the risk of macrovascular complications among 14,277 UK Biobank participants.

**Results:**

Compared with non-coffee or tea drinkers, those who consumed 0.5–1 cup of coffee (HR 0.67,95% CI 0.518 to 0.856) or 2–4 cups of tea (HR 0.66,95% CI 0.524 to 0.839) per day had the lowest risk of stroke; daily intake of 2–4 cups of coffee associated with reduced risk of angina pectoris (AP) (HR 0.82,95% CI 0.726 to 0.916); those who consumed 0.5–1 cup of tea per day had the lowest risk of the heart failure (HF) (HR 0.73,95% CI 0.602 to 0.879); furthermore, those who consumed 2–4 cups of coffee and 0.5–1 cup of tea per day (HR 0.55, 95% CI 0.379–0.790) demonstrated the lowest risk of HF onset compared with those who did not consume coffee and tea at all.

**Conclusions:**

This study found that in a T2DM population, moderate coffee consumption significantly lowered the risk of stroke and AP, while moderate tea intake reduced the risk of stroke and HF. Combined moderate consumption of both beverages provided optimal protection against HF.

**Supplementary Information:**

The online version contains supplementary material available at 10.1186/s13098-025-01807-4.

## Introduction

Type 2 diabetes mellitus (T2DM) is a growing global public health problem, with an estimated 537 million people living with it by 2021 [1]. Macrovascular complications such as stroke, angina pectoris (AP) and, heart failure (HF) are common comorbidities in people with diabetes, leading to reduced quality of life and premature death [2]. Identifying modifiable risk factors is essential to prevent or delay the onset of macrovascular complications in patients with T2DM.

Caffeine and antioxidants are active ingredients in coffee, tea and other beverages with protective effects against macrovascular disease [3]. Consequently, highly caffeinated beverages, such as coffee and tea, are currently attracting significant interest in the field of macrovascular disease prevention. In the general population, some studies have found that the type and amount of coffee and tea consumed is significantly associated with the risk of cardiovascular disease [4–6]. For example, a meta-analysis included 11 cohort studies [7], of which 9 were conducted in Western countries [8–18] and 2 in Asian countries [19, 20], reported that consumption of one to three cups of tea per day reduced the risk of stroke by 14%. Another study found that moderate daily coffee intake was associated with a reduced risk of cardiovascular disease [21]. Epidemiologic studies have shown that green tea consumption is associated with a reduced risk of cardiovascular disease [22], but there is little evidence on the role of other teas and their combination with coffee in diabetic populations. Although the mechanism has not been fully elucidated, experts agree that components of coffee and tea, such as caffeine, can reduce the risk of macrovascular disease by lowering blood cholesterol and triacylglycerol levels, causing blood vessels to constrict, and enhancing the excitability of the central nervous system, which improves myocardial contraction to some extent [23, 24]. In diabetic populations, some studies have suggested that bioactive substances such as catechins and caffeine in coffee and tea may reduce the risk of diabetes by enhancing insulin activity, improving insulin resistance, and reducing inflammation [25–28]. For instance, Shahinfar et al. [29] demonstrated that moderate coffee consumption was associated with a lower risk of cardiovascular mortality in patients with T2DM, while Ma et al. [30] reported similar protective effects for tea. However, these studies primarily focused on individual beverage consumption, leaving the combined effects unexplored. To date, research on the relationship between coffee and tea consumption and risk of macrovascular disease in people with T2DM remains limited, and it is unclear whether the benefits of these two beverages for T2DM apply equally to people with T2DM-related vascular complications, most people still have limited knowledge of the association between coffee and tea consumption and macrovascular complications due to diabetes. Additionally, while the individual effects of coffee or tea on macrovascular complications have been explored, their combined consumption and potential synergistic or additive effects remain unclear. Given that many individuals consume both beverages regularly, investigating their joint impact may provide more comprehensive dietary recommendations for patients with T2DM.

To fill these gaps, this study utilized data from the UK Biobank to investigate the relationship between coffee and tea intake and the risk of macrovascular complications in patients with T2DM, with the aim of providing a scientific basis for dietary management and prevention of macrovascular complications in patients with T2DM.

## Methods

### Study population

**Fig. 1:**
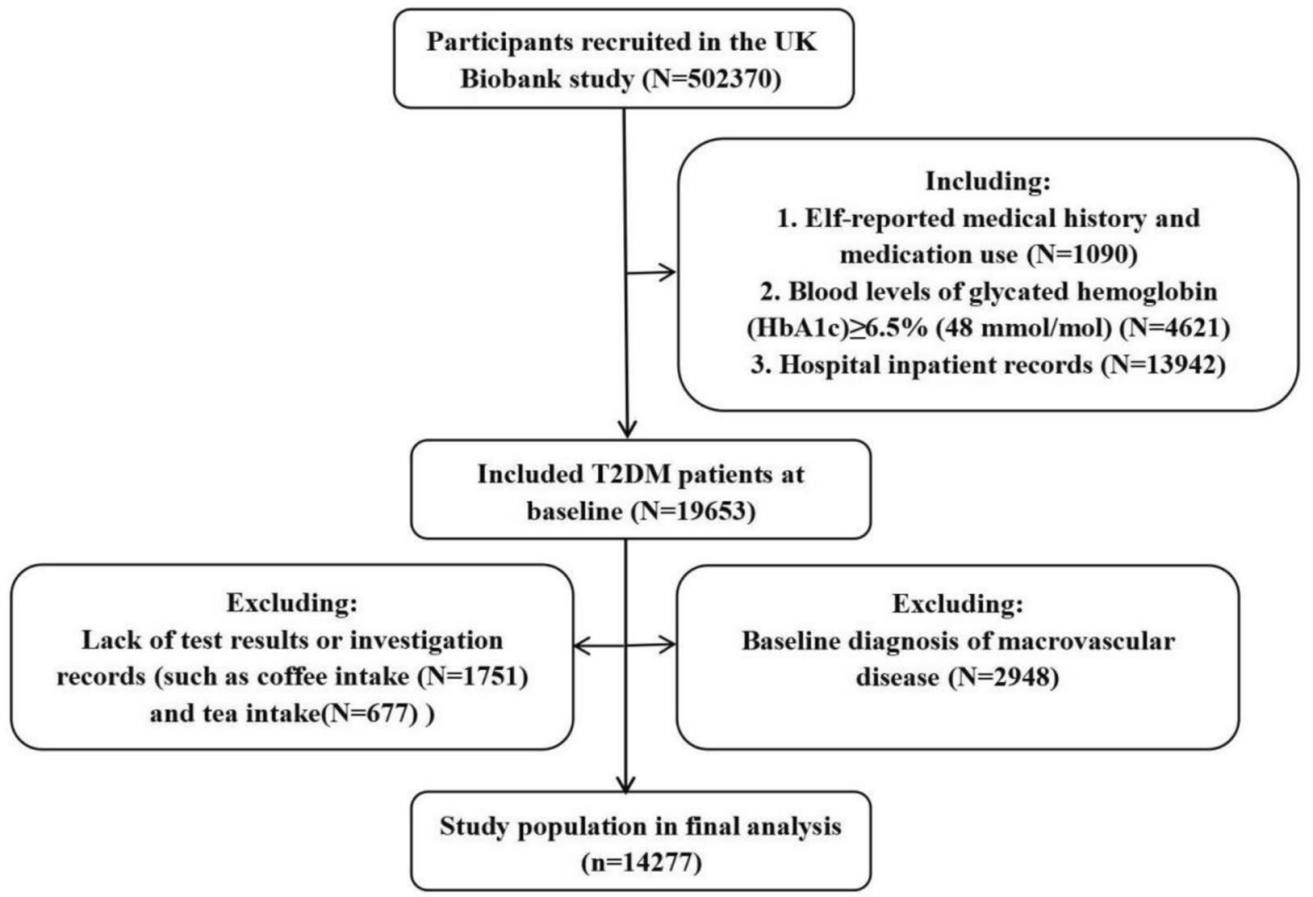
Flowchart of participants included in the analysis

The UK Biobank is a vast population-based study that recruited more than 500,000 participants (aged 39–74) at 22 assessment centers in England, Wales, and Scotland from 2006 to 2010 [31]. Participants underwent physical examinations conducted by trained staff and completed touchscreen questionnaires. The cohort ensures continuous tracking of health-related outcome data of participant by establishing links with electronic records from primary care, hospitalization, and death registries. It has gathered a broad array of genetic and health data to explore genetic and lifestyle influences on various common diseases in middle-aged and older adults [32]. All participants signed the informed consent form. In addition, UK Biobank adheres to the ethical principles of the 1975 Helsinki Declaration and has been approved by the North West Multi-Centre Research Ethics Committee (MREC) as a Research Tissue Bank (RTB), enabling researchers to operate within the approved scope without requiring additional ethical clearance. Among 502,370 UK Biobank participants collected at baseline, patients with T2DM at baseline were considered potentially eligible participants. As reported elsewhere [33], T2DM was considered present according to one or more of the following 3 diagnostic criteria: (1) self-reported medical history and medication use, (2) blood levels of glycated hemoglobin (HbA1c) ≥ 6.5% (48 mmol/mol), (3) hospital inpatient records. Those with any of the following conditions were also excluded: (1) lack of test results or investigation records, (2) baseline diagnosis of macrovascular disease. Ultimately, 14,277 participants were included in this study, with follow-up until December 1, 2023, and an average follow-up time of 14.40 years. Study participant flow based on inclusion and exclusion criteria is reported in Fig. 1.

### Exposure assessment

A preliminary survey conducted by the UK Biobank collected data on coffee and tea consumption through the Food Frequency Questionnaire (FFQ), with the objective of assessing the type and intake of coffee and tea [34]. The FFQ was administered at baseline (2006–2010) and captured habitual consumption patterns over the preceding year. In this study, “tea consumption” refers to the consumption of any type of tea, including black tea, green tea; “coffee consumption” refers to the consumption of any type of coffee, including ground coffee, instant coffee, and decaffeinated coffee. Participants were requested to provide the number of cups of coffee and tea consumed per day (participants chose one of the following options: “Less than one”, “Don’t know”, “Prefer not to answer,”, or the daily specific number of cups. If participants reported drinking more than 10 cups per day, they were asked to confirm their response), as well as the types of coffee typically consumed (e.g., “Decaffeinated coffee,” “Instant coffee,” “Ground coffee, “Do not know,” or “prefer not to answer.”).

### Ascertainment of outcomes

Outcomes were ascertained using hospital inpatient records containing data on admissions and diagnoses obtained from the Hospital Episode Statistics for England, the Scottish Morbidity Record data for Scotland, and the Patient Episode Database for Wales. Diagnoses were recorded using the International Classification of Diseases-10th revision (ICD-10) coding system. The primary outcomes in this study were incident of stroke, AP and HF. Incident cases were defined as the first occurrence of a macrovascular complication (stroke, AP, or HF) recorded in the inpatient databases during follow-up. This approach captures the earliest clinical diagnosis documented in the healthcare system. The definitions of them in the UK biobank are provided in Table S1.

### Assessment of other covariates

The baseline survey obtained potential confounders such as corresponding socio-demographic, lifestyle and health-related issues through questionnaires. Confounders included age, sex, ethnicity (White, Asian, Black, and Other ethnic group), qualification (Equivalent to or less than high school diploma, college or university degree, A level/AS levels or equivalent, O level/GCSEs or equivalent, CSEs or equivalent, NVQ or HND or HNC or equivalent, and other professional qualifications), income (less than £18,000, 18,000 to 30,999, 31,000 to 51,999, 52,000 to 100,000,and greater than 100,000), body mass index (BMI)(< 25, 25 to < 30, 30 to < 35, and ≥ 35 kg/m^2^), smoking status (never, former, and current), alcohol status (never, former, and current), physical activity (low, moderate, and high), high-density lipoprotein (HDL), low-density lipoprotein(LDL), and diet pattern (healthy and unhealthy, healthy diet was based on consumption of at least 4 of 7 dietary components: (1) fruits: ≥ 3 servings/day; (2) vegetables: ≥ 3 servings/day; (3) fish: ≥ 2 servings/week; (4) processed meats: ≤ 1 serving/week; (5) unprocessed red meats: ≤ 1.5 servings/week; (6) whole grains: ≥ 3 servings/day; (7) refined grains: ≤ 1.5 servings/day) (Table S2). The cutoff of ≥ 4 components was chosen to define a healthy diet pattern based on prior studies linking this threshold to reduced cardiovascular risk [35, 36]. Baseline hypertension was defined as systolic blood pressure ≥ 140 mmHg, diastolic blood pressure ≥ 90 mmHg. Duration of T2DM was calculated by subtracting a participant’s age at diagnosis of T2DM from the age at baseline interview [37].

### Statistical analyses

The primary analysis examined the relationship between coffee and tea intake (assessed via baseline FFQ) and the risk of macrovascular complications in individuals with T2DM using Cox proportional hazards models. All statistical analyses were performed using Stata software version 17 and R version 4.0.2. In descriptive analyses, one-way ANOVA determined the mean [standard deviation (SD)] of continuous variables between groups, whereas the Pearson χ^2^ test determined any statistical differences in proportions of categorical variables. Cox regression models were used to estimate hazard ratios (HR) and 95% confidence intervals (CI) of stroke, AP and HF associated with different levels of coffee consumption and tea consumption, using 0 cups per day as the reference group. Person-time of follow-up was calculated as the duration from the date of baseline evaluation until the date of the diagnosis of macrovascular complications, death, loss to follow-up, or end of follow-up, whichever occurred first. Cox regression models were adjusted for sex, age, ethnicity, qualification, income, BMI, physical activity, alcohol status, smoking status, HDL, LDL, diet pattern, and hypertension.

Restricted cubic spline models were used to evaluate the relationship between coffee, tea, and their combination and incident stroke, AP and HF with 4 knots at the 25th, 50th, 75th, and 95th centiles. In the spline models, we adjusted for sex, age, ethnicity, Qualification, income, BMI, physical activity, alcohol status, smoking status, diet pattern, HDL, LDL and hypertension.

To evaluate potential effect modification by sex, smoking status, BMI, and physical activity on the association between coffee/tea consumption and macrovascular complications, we conducted stratified analyses by these variables. In addition, to assess the robustness of the findings, we conducted sensitivity analyses by excluding participants who developed macrovascular disease within the first 2 years of follow-up, thereby reducing the potential for reverse causality in the observed associations. All P-values were 2 sided, with statistical significance set at less than 0.05.

## Results

### Baseline characteristics of the participants

Table 1 displays the baseline characteristics of 14,277 participants stratified by the presence of incident macrovascular complications. The mean age of the 14,277 patients with T2DM was 59.31 ± 7.16 years. Over a median follow-up period of 14.40 years, 3095 individuals (21.7%) developed macrovascular complications. Among participants with T2DM, a higher incidence of macrovascular complications were more likely to be found in male, Asian or Black population, participants with low education and income, high BMI, unhealthy diet, current smoking, former alcohol use, low physical activity, concurrent hypertension, and non-moderate coffee or tea consumption. Additionally, the participants with macrovascular complications had lower baseline level of HDL.

**Table 1:** Baseline characteristics of participants by macrovascular complication status

Characteristic	Total population (N = 14,277)	Participants without Macrovascular complications (N = 11,182)	Participants with Macrovascular complications (N = 3095)	χ^2^/F	P value
Mean age at baseline (SD)	59.31 (7.16)	58.58 (7.30)	61.92 (5.98)	26.10	< 0.001
Age at the end of follow-up, years, mean (SD)	75.17 (7.20)	74.42 (7.32)	77.87 (6.02)	578.786	< 0.001
Sex, n (%)				90.794	< 0.001
Female	4849 (33.96)	4020 (82.90)	829 (17.10)		
Male	9428 (66.04)	7162 (75.97)	2266 (24.03)		
Ethnicity, n (%)				11.668	0.009
White	275 (1.93)	227 (82.55)	48 (17.45)		
Asian	12,864 (90.10)	10,042 (78.06)	2822 (21.94)		
Black	733 (5.13)	572 (78.04)	161 (21.96)		
Other	405 (2.84)	341 (84.20)	64 (15.80)		
Qualification, n (%)				243.58	< 0.001
Equivalent to or less than high school diploma	3008 (21.07)	2073 (68.92)	935 (31.08)		
College or University	4130 (28.93)	3452 (83.58)	678 (16.42)		
A level/AS levels or equivalent	1469 (10.29)	1200 (81.69)	269 (18.31)		
O level/GCSEs or equivalent	2940 (20.59)	2345 (79.76)	595 (20.24)		
CSEs or equivalent	655 (4.59)	524 (80.00)	131 (20.00)		
NVQ or HND or HNC or equivalent	1250 (8.76)	947 (75.76)	303 (24.24)		
Other professional qualifications eg: nursing, teaching	825 (5.78)	641 (77.70)	184 (22.30)		
Income, n (%)				427.644	< 0.001
Less than 18,000	4822 (33.77)	3355 (69.58)	1467 (30.42)		
18,000–30999	4059 (28.43)	3182 (78.39)	877 (21.61)		
31,000–51999	3043 (21.31)	2565 (84.29)	478 (15.71)		
52,000 to 10,000	1945 (13.62)	1701 (87.46)	244 (12.54)		
Greater than 10,000	408 (2.86)	379 (92.89)	29 (7.11)		
BMI (kg/m^2^), n (%)				190.197	< 0.001
< 25	1702 (11.92)	1468 (86.25)	234 (13.75)		
25–30	4943 (34.62)	4037 (81.67)	906 (18.33)		
30–35	4267 (29.89)	3263 (76.47)	1004 (23.53)		
≥ 35	3365 (23.57)	2414 (71.74)	951 (28.26)		
Diet, n (%)				9.853	0.002
Unhealthy	9851 (69.00)	7644 (77.60)	2207 (22.40)		
Healthy	4426 (31.00)	3538 (79.94)	888 (20.06)		
Smoking status, n (%)				144.681	< 0.001
Never	6387 (44.74)	5296 (82.92)	1091 (17.08)		
Former	6325 (44.30)	4732 (74.81)	1593 (25.19)		
Current	1565 (10.96)	1154 (73.74)	411 (26.26)		
Alcohol status, n (%)				62.900	< 0.001
Never	924 (6.47)	700 (75.76)	224 (24.24)		
Former	991 (6.94)	682 (68.82)	309 (31.18)		
Current	12,362 (86.59)	9800 (79.28)	2562 (20.72)		
Physical activity, n (%)				94.707	< 0.001
Low	3911 (27.39)	2851 (72.90)	1060 (27.10)		
Moderate	6846 (47.95)	5479 (80.03)	1367 (19.97)		
High	3520 (24.66)	2852 (81.02)	668 (18.98)		
Hypertension, n (%)				247.382	< 0.001
No	4285 (30.01)	3711 (86.60)	574 (13.40)		
Yes	9992 (69.99)	7471 (74.77)	2521 (25.23)		
HDL, mmol/L, mean (SD)	1.21 (0.30)	1.22 (0.31)	1.14 (0.29)	197.986	< 0.001
LDL, mmol/L, mean (SD)	2.69 (0.73)	2.71 (0.73)	2.63 (0.71)	26.338	< 0.001
Coffee intake, median (IQR), cups/day	2 (1.0–2.0)	2 (1.0—2.0)	2 (1.0–2.0)	0.828	0.407
Coffee intake, n (%)				12.740	0.005
0	3232 (22.64)	2479 (76.70)	753 (23.30)		
0.5–1	3717 (26.03)	2943 (79.18)	774 (20.82)		
2–4	5486 (38.43)	4349 (79.27)	1137 (20.73)		
≥ 5	1842 (12.90)	1411 (76.60)	431 (23.40)		
Tea intake, median (IQR), cups/day	2 (1.0–3.0)	2 (1.0–3.0)	2 (1.0–3.0)	0.460	0.645
Tea intake, n (%)				18.670	< 0.001
0	2599 (18.20)	1984 (76.34)	615 (23.66)		
0.5–1	1765 (12.36)	1430 (81.02)	335 (18.98)		
2–4	5958 (41.73)	4714 (79.12)	1244 (20.88)		
≥ 5	3955 (27.70)	3054 (77.22)	901 (22.78)		

Date are n (%)

*A* Advanced, *AS* Advanced Subsidiary, *BMI* body mass index, *CSE* Certificate of Secondary Education, *GCSE* General Certificate of Secondary Education, *HDL* high-density lipoprotein, *HNC* Higher National Certificate, *HND* Higher National Diploma, *LDL* low-density lipoprotein, *NVQ* National Vocational Qualification, *O* Ordinary, *SD* standard deviation

### The risk of stroke associated with the consumption of coffee and tea

**Fig. 2:**
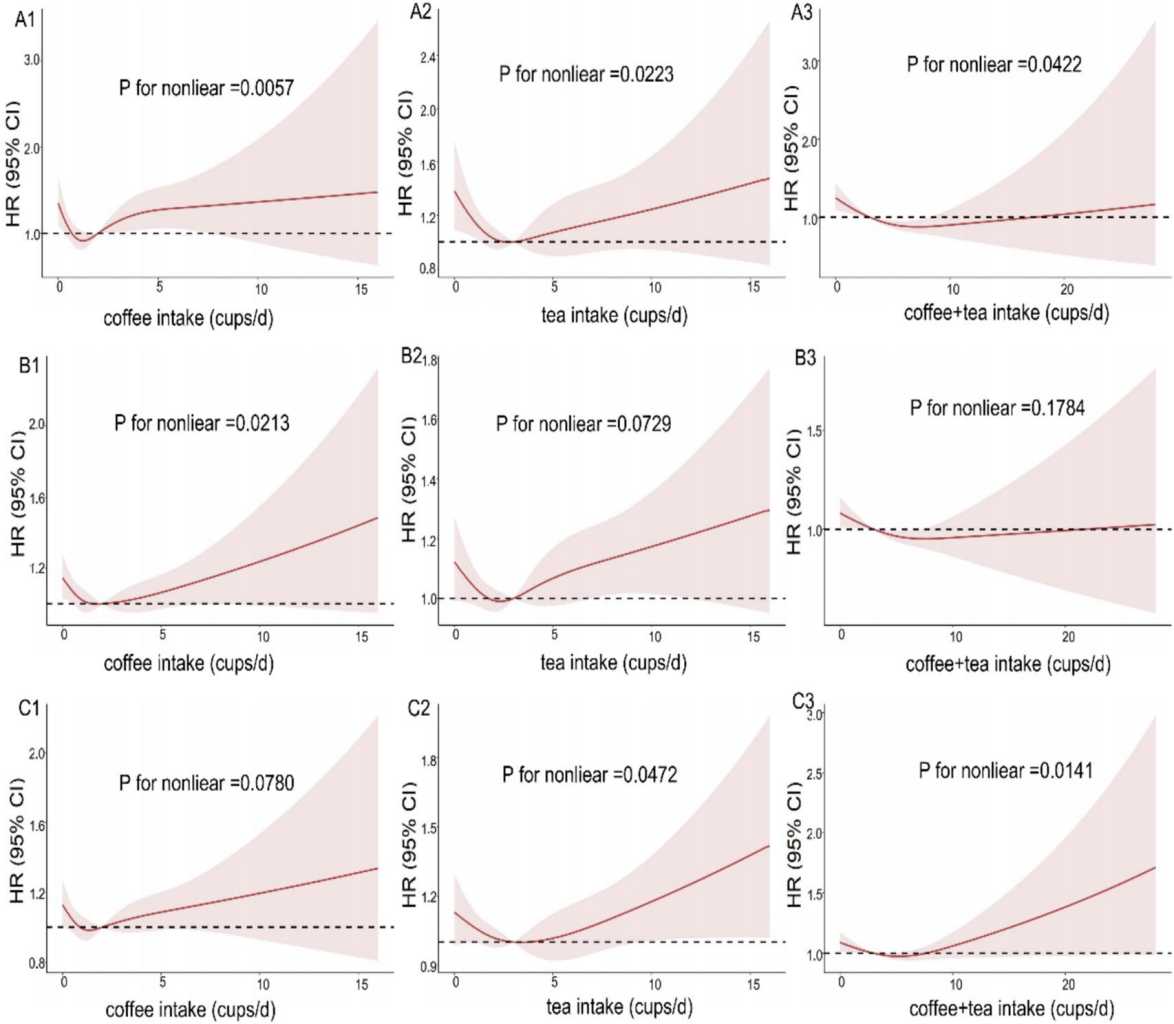
Restricted cubic spline models for the relationship between coffee, tea, and their combination with stroke, AP, and HF. **A1** Coffee and stroke. **A2** Tea and stroke. **A3** Combination of coffee and tea on stroke. **B1** Coffee and AP. **B2** Tea and AP. **B3** Combination of coffee and tea on AP. **C1** Coffee and HF. **C2** Tea and HF. **C3** Combination of coffee and tea on HF. The 95% CI of the adjusted HR are represented by the shaded area. Restricted cubic spline model is adjusted for sex, age, ethnicity, qualification, income, BMI, smoking status, alcohol status, physical activity, diet, HDL, LDL, hypertension and we adjusted for coffee in tea analysis or for tea in coffee analysis. *CI* confidence interval, *HR* hazard ratio, *HF* heart failure, *BMI* body mass index, *HDL* high-density lipoprotein, *LDL* low-density lipoprotein, *AP* angina pectoris, *HF* heart failure

**Fig. 3:**
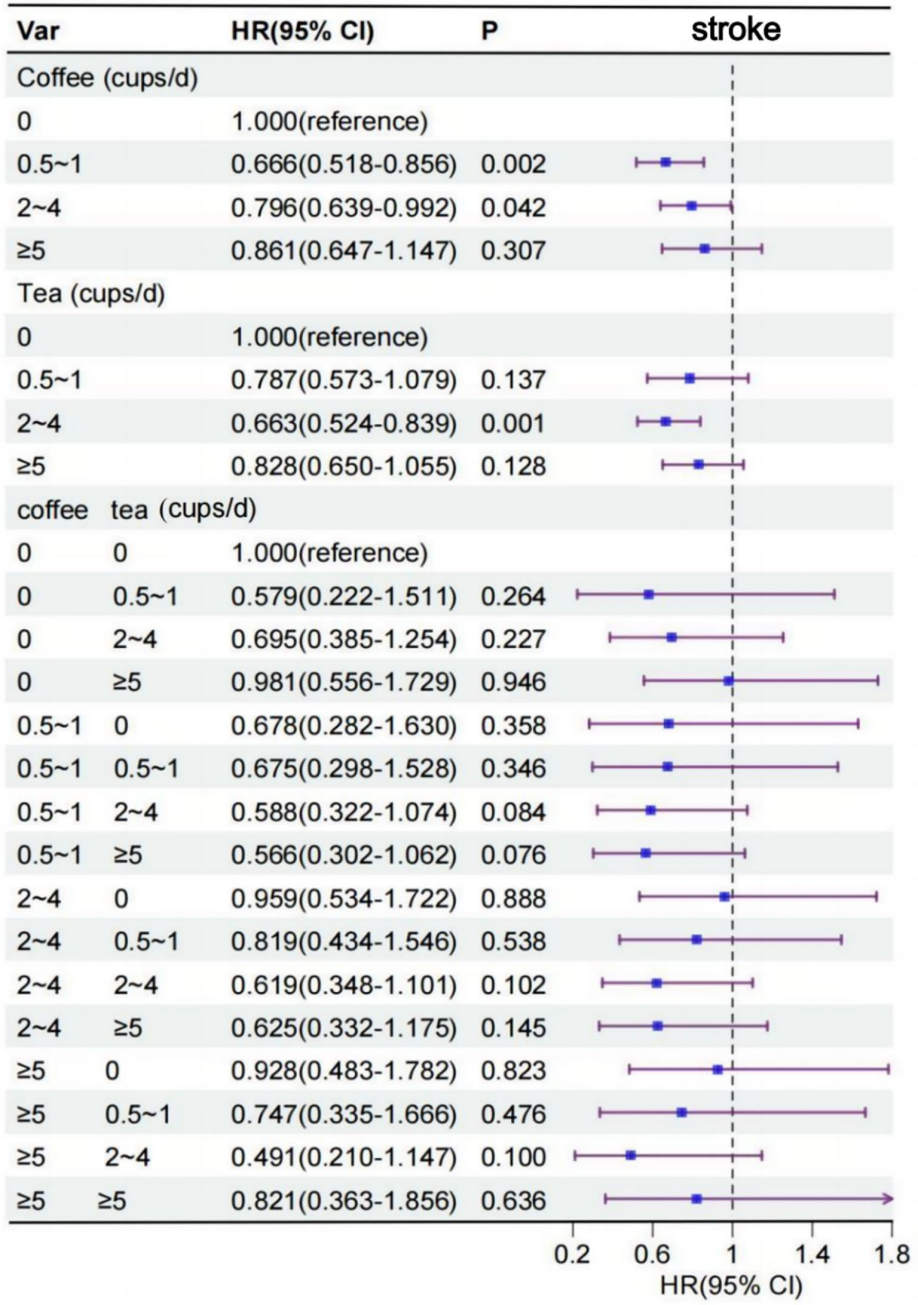
Association of coffee and tea intake with stroke. Multivariable model is adjusted for sex, age, ethnicity, qualification, income, BMI, smoking status, alcohol status, physical activity, diet pattern, HDL, LDL, hypertension and we adjusted for coffee in tea analysis or for tea in coffee analysis. *CI* confidence interval, *HR* hazard ratio, *BMI* body mass index, *HDL* high-density lipoprotein, *LDL* low-density lipoprotein

To analyze the association between coffee and tea intake and new onset outcomes, we defined coffee and tea intake into the following categories: 0, 0.5 to 1, 2 to 4, and ≥ 5 cups/day. To assess the associations between coffee, tea, and their combinations with stroke, AP, and HF, both unadjusted (Figure S1) and multivariable-adjusted (Fig. 2) restricted cubic spline models were employed. After adjustment for all covariates, a J-shaped relationship was observed between coffee consumption, tea consumption, and consumption of the combination of the two, respectively, and stroke risk. We investigated the association of each coffee and tea intake with stroke (Fig. 3). In unadjusted Cox models, coffee and tea intakes were associated with lower risk of stroke (Table S3). After multivariable adjustment, coffee intake was associated with lower risk of stroke. Compared to that of noncoffee drinkers, HR (95% CI) for coffee intake of 0.5 to 1, 2 to 4 cups/d were 0.67 (95% CI 0.518 to 0.856; P = 0.002) and 0.80 (95% CI 0.639 to 0.992; P = 0.042), respectively. Likewise, after multivariable adjustment for confounding factors, tea intake was associated with lower risk of stroke. HR (95% CI) of stroke for tea intake of 2 to 4 cups/d were 0.66 (95% CI 0.524 to 0.839; P = 0.001) (Fig. 3). In addition, we evaluated the association of coffee type with stroke and showed that ground coffee was associated with a lower risk of stroke compared with decaffeinated coffee (HR, 0.70; 95% CI 0.491 to 0.999; P = 0.049) (Table S4).

### The risk of AP associated with the consumption of coffee and tea

**Fig. 4:**
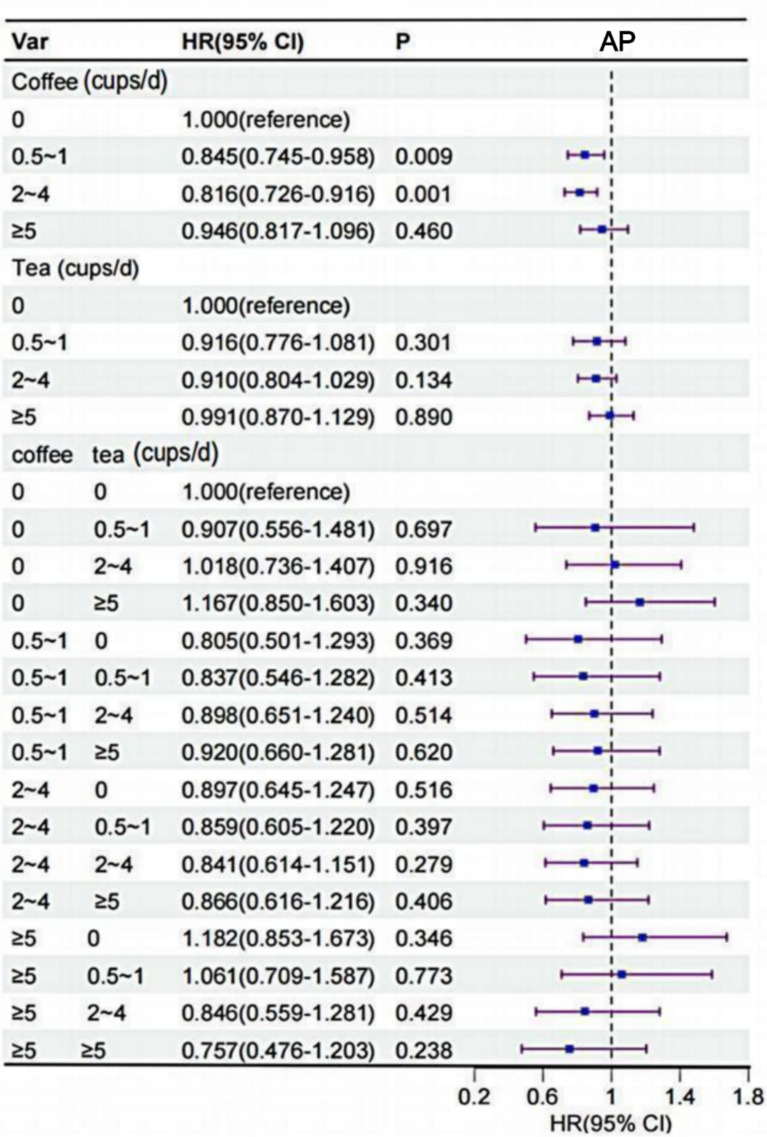
Association of coffee and tea intake with AP. Multivariable model is adjusted for sex, age, ethnicity, qualification, income, BMI, smoking status, alcohol status, physical activity, diet pattern, HDL, LDL, hypertension and we adjusted for coffee in tea analysis or for tea in coffee analysis. *CI* confidence interval, *HR* hazard ratio, *AP* angina pectoris, *BMI* body mass index, *HDL* high-density lipoprotein, *LDL* low-density lipoprotein

Following the adjustment for all covariates, a J-shaped relationship was observed between coffee consumption and AP risk (Fig. 2). Subsequently, we assessed the association of each coffee and tea with AP (Fig. 4). In unadjusted Cox models, intake of coffee was associated with lower risk of AP (Table S3). After multivariable adjustment for confounding factors, coffee intake was associated with lower risk of AP. Compared to that of noncoffee drinkers, HR (95% CI) for coffee intake of 0.5 to 1 and 2 to 4 cups/d were 0.85 (95% CI 0.745 to 0.958; P = 0.009) and 0.82 (95% CI 0.726 to 0.916; P = 0.001), respectively. In addition, compared to nontea drinking, tea intake was not associated with AP (Fig. 4). Results based on the association of coffee type with AP showed that instant coffee was associated with a lower risk of AP compared with decaffeinated coffee (HR, 0.87; 95% CI 0.763 to 0.994; P = 0.041) (Table S4).

### The risk of HF associated with the consumption of coffee and tea

**Fig. 5:**
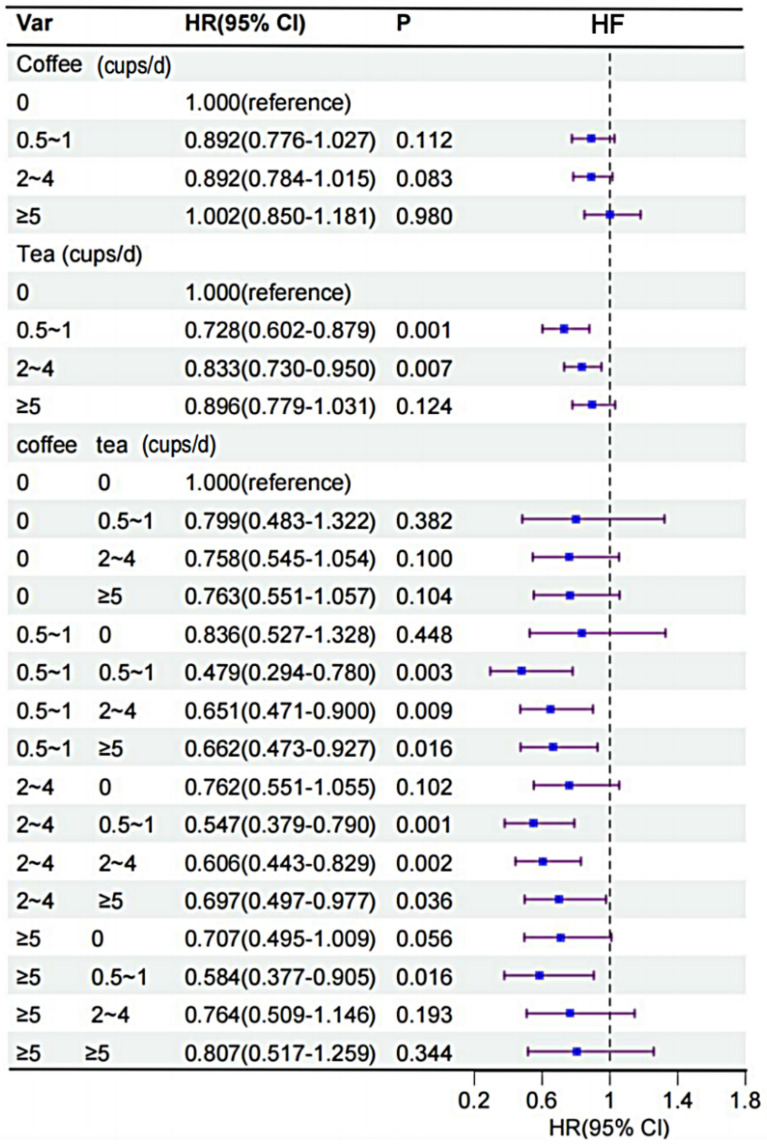
Association of coffee and tea intake with HF. Multivariable model is adjusted for sex, age, ethnicity, qualification, income, BMI, smoking status, alcohol status, physical activity, diet pattern, HDL, LDL, hypertension and we adjusted for coffee in tea analysis or for tea in coffee analysis. *CI* confidence interval, *HR* hazard ratio, *HF* heart failure, *BMI* body mass index, *HDL* high-density lipoprotein, *LDL* low-density lipoprotein

Following the adjustment for all covariates, a J-shaped relationship was observed between tea consumption and the consumption of a combination of coffee and tea and the risk of developing HF (Fig. 2). We further studied the association of coffee and tea with HF (Fig. 5). In unadjusted Cox models, tea and the combination of coffee and tea were associated with lower risk of HF (Table S3). After multivariable adjustment, participants with a daily intake of 0.5 to 1 cup of tea had a lower risk of heart failure (HR, 0.73; 95% CI, 0.602 to 0.879; P = 0.001). In addition, compared to noncoffee drinking, coffee intake was not associated with HF. Next, we assessed the combination of coffee and tea intake on HF. We found that the combination of coffee and tea was associated with lower risk of HF. Compared with those who did not drink coffee and tea, HR of drinking 2 to 4 cup of coffee and 0.5 to 1 cups of tea per day were 0.55 (95% CI 0.379 to 0.790, P = 0.001) for HF (Fig. 5).

### Subgroup analysis and sensitivity analysis

In the analysis of the association between macrovascular complications in T2DM and coffee, tea, and their combinations, stratified analysis was conducted by considering sex, smoking status, BMI and physical activity. When analyses were stratified by smoking status, the association between tea intake and risk of AP was more pronounced in patients with T2DM who drank 2–4 cups of tea per day (P for interaction = 0.021; Table 6), but not in stroke and HF (P for interaction = 0.731; P for interaction = 0.418; Tables 5, 7). The association of coffee intake with stroke, AP, and HF did not differ significantly (all P for interaction > 0.05) with respect to sex (Tables 2, 3, 4), smoking status (Tables 5, 6, 7), physical activity (Figures S2–S4), and BMI (Figures S5–S7). We conducted a sensitivity analysis by excluding participants who developed macrovascular disease within the first 2 years of follow-up, and all associations remained significant and consistent with the overall study results, as shown in Table S5, which demonstrates the robustness of the findings.

**Table 2:** Association of coffee and tea with stroke in the UK Biobank cohort by sex

Group		Women				Men				P for interaction
		Unadjusted HR (95% CI)	*P* value	Multi-adjusted HR (95% CI)	*P* value	Unadjusted HR (95% CI)	*P* value	Multi-adjusted HR (95% CI)	*P* value	
Coffee (cups/d)										
0		1.000 (Ref)		1.000 (Ref)		1.000 (Ref)		1.000 (Ref)		0.524
0.5–1		0.599 (0.375–0.957)	0.032	0.624 (0.387–1.004)	0.052	0.629 (0.469–0.844)	0.002	0.681 (0.505–0.916)	0.011	
2–4		0.754 (0.499–1.140)	0.181	0.752 (0.492–1.150)	0.189	0.735 (0.571–0.947)	0.017	0.818 (0.631–1.060)	0.129	
≥ 5		0.669 (0.353–1.267)	0.218	0.594 (0.310–1.137)	0.116	0.852 (0.620–1.172)	0.326	0.952 (0.687–1.320)	0.770	
Tea (cups/d)										
0		1.000 (Ref)		1.000 (Ref)		1.000 (Ref)		1.000 (Ref)		0.134
0.5–1		1.220 (0.689–2.158)	0.495	1.340 (0.755–2.380)	0.317	0.600 (0.409–0.880)	0.009	0.630 (0.429–0.925)	0.019	
2–4		0.754 (0.465–1.224)	0.253	0.770 (0.472–1.255)	0.294	0.670 (0.514–0.873)	0.003	0.627 (0.479–0.820)	<0.001	
≥ 5		1.125 (0.692–1.830)	0.634	1.177 (0.721–1.923)	0.515	0.797 (0.605–1.051)	0.108	0.726 (0.549–0.959)	0.024	
Coffee (cups/d)	Tea (cups/d)									
0	0	1.000 (Ref)		1.000 (Ref)		1.000 (Ref)		1.000 (Ref)		0.282
0	0.5–1	0.830 (0.215–3.212)	0.788	0.727 (0.182–2.911)	0.653	0.699 (0.185–2.636)	0.597	0.452 (0.117–1.739)	0.248	
0	2–4	0.438 (0.163–1.175)	0.101	0.347 (0.127–0.947)	0.039	1.276 (0.596–2.730)	0.530	0.909 (0.419–1.973)	0.809	
0	≥ 5	1.264 (0.546–2.922)	0.585	1.092 (0.465–2.564)	0.840	1.366 (0.649–2.876)	0.411	0.935 (0.435–2.010)	0.863	
0.5–1	0	0.610 (0.155–2.400)	0.479	0.557 (0.130–2.377)	0.429	0.906 (0.296–2.771)	0.863	0.825 (0.263–2.582)	0.740	
0.5–1	0.5–1	0.913 (0.290–2.879)	0.877	1.062 (0.327–3.448)	0.921	0.551 (0.180–1.684)	0.296	0.519 (0.166–1.623)	0.260	
0.5–1	2–4	0.413 (0.159–1.069)	0.068	0.431 (0.161–1.152)	0.093	0.832 (0.390–1.773)	0.633	0.669 (0.303–1.476)	0.320	
0.5–1	≥5	0.494 (0.188–1.299)	0.153	0.533 (0.192–1.479)	0.227	0.727 (0.330–1.600)	0.428	0.601 (0.264–1.369)	0.225	
2–4	0	0.665 (0.258–1.719)	0.400	0.621 (0.235–1.641)	0.336	1.132 (0.514–2.492)	0.759	1.385 (0.617–3.111)	0.430	
2–4	0.5–1	0.772 (0.287–2.073)	0.607	0.752 (0.273–2.072)	0.581	1.524 (0.942–2.467)	0.086	1.699 (1.046–2.758)	0.032	
2–4	2–4	0.711 (0.302–1.675)	0.436	0.703 (0.287–1.723)	0.441	1.030 (0.582–1.822)	0.920	1.263 (0.710–2.245)	0.426	
2–4	≥ 5	0.476 (0.151–1.501)	0.205	0.499 (0.155–1.611)	0.499	0.843 (0.531–1.337)	0.467	0.891 (0.559–1.419)	0.626	
≥ 5	0	0.571 (0.192–1.699)	0.314	0.487 (0.149–1.587)	0.232	1.411 (0.651–3.055)	0.383	1.352 (0.580–3.152)	0.485	
≥ 5	0.5–1	1.028 (0.301–3.516)	0.964	1.860 (0.292–4.038)	0.902	0.720 (0.270–1.920)	0.512	0.821 (0.289–2.337)	0.712	
≥ 5	2–4	0.260 (0.302–2.112)	0.207	0.182 (0.200–1.692)	0.134	0.670 (0.258–1.736)	0.410	0.721 (0.261–1.991)	0.528	
≥ 5	≥ 5	0.392 (0.048–3.184)	0.381	0.476 (0.056–4.058)	0.497	1.308 (0.516–3.314)	0.572	1.182 (0.443–3.151)	0.738	

Multivariable model is adjusted for age, ethnicity, qualification, income, BMI, smoking status, alcohol status, physical activity, diet pattern, HDL, LDL, hypertension and we adjusted for coffee in tea analysis or for tea in coffee analysis

*CI* confidence interval, *HR* hazard ratio, *UK Biobank* United Kingdom Biobank, *BMI* body mass index, *HDL* high-density lipoprotein, *LDL* low-density lipoprotein

**Table 3:** Association of coffee and tea with AP in the UK Biobank cohort by sex

Group		Women				Men				P for interaction
		Unadjusted HR (95% CI)	*P* value	Multi-adjusted HR (95% CI)	*P* value	Unadjusted HR (95% CI)	*P* value	Multi-adjusted HR (95% CI)	*P* value	
Coffee (cups/d)										
0		1.000 (Ref)		1.000 (Ref)		1.000 (Ref)		1.000 (Ref)		0.483
0.5–1		0.736 (0.582–0.930)	0.010	0.746 (0.589–0.947)	0.016	0.841 (0.726–0.975)	0.022	0.898 (0.773–1.043)	0.157	
2–4		0.745 (0.598–0.928)	0.009	0.738 (0.590–0.924)	0.008	0.801 (0.700–0.916)	0.001	0.895 (0.747–0.983)	0.027	
≥ 5		1.075 (0.809–1.430)	0.618	0.946 (0.707–1.267)	0.710	0.902 (0.762–1.068)	0.233	0.971 (0.871–1.154)	0.793	
Tea (cups/d)										
0		1.000 (Ref)		1.000 (Ref)		1.000 (Ref)		1.000 (Ref)		0.710
0.5–1		0.986 (0.723–1.344)	0.929	1.056 (0.773–1.442)	0.732	0.808 (0.665–0.982)	0.032	0.871 (0.716–1.059)	0.167	
2–4		0.934 (0.737–1.184)	0.523	0.977 (0.769–1.242)	0.848	0.891 (0.773–1.027)	0.111	0.886 (0.767–1.023)	0.098	
≥ 5		1.040 (0.808–1.338)	0.761	1.132 (0.878–1.459)	0.340	0.957 (0.823–1.112)	0.562	0.945 (0.812–1.100)	0.467	
Coffee (cups/d)	Tea (cups/d)									
0	0	1.000 (Ref)		1.000 (Ref)		1.000 (Ref)		1.000 (Ref)		0.822
0	0.5–1	1.528 (0.678–3.440)	0.306	1.372 (0.601–3.134)	0.452	0.783 (0.426–1.438)	0.430	0.710 (0.383–1.315)	0.276	
0	2–4	1.670 (0.935–2.981)	0.083	1.514 (0.839–2.732)	0.168	0.941 (0.645–1.373)	0.752	0.817 (0.554–1.205)	0.308	
0	≥ 5	1.829 (1.029–3.249)	0.040	1.776 (0.992–3.179)	0.053	1.029 (0.712–1.487)	0.878	0.928 (0.636–1.355)	0.699	
0.5–1	0	1.146 (0.509–2.581)	0.742	0.951 (0.419–2.161)	0.905	0.714 (0.399–1.279)	0.257	0.815 (0.452–1.469)	0.496	
0.5–1	0.5–1	1.202 (0.565–2.557)	0.633	1.041 (0.483–2.245)	0.918	0.683 (0.412–1.132)	0.139	0.765 (0.457–1.282)	0.309	
0.5–1	2–4	1.191 (0.666–2.129)	0.555	0.985 (0.542–1.792)	0.961	0.862 (0.597–1.243)	0.426	0.874 (0.596–1.283)	0.492	
0.5–1	≥ 5	1.155 (0.632–2.111)	0.640	0.977 (0.524–1.825)	0.943	0.856 (0.587–1.248)	0.418	0.891 (0.601–1.321)	0.565	
2–4	0	1.286 (0.702–2.356)	0.415	1.095 (0.590–2.030)	0.774	1.232 (0.838–1.811)	0.290	1.253 (0.843–1.863)	0.265	
2–4	0.5–1	1.432 (0.764–2.685)	0.262	1.339 (0.707–2.538)	0.371	1.031 (0.794–1.338)	0.821	1.010 (0.776–1.314)	0.941	
2–4	2–4	1.108 (0.622–1.973)	0.728	0.966 (0.531–1.756)	0.909	0.830 (0.614–1.120)	0.223	0.894 (0.661–1.210)	0.468	
2–4	≥ 5	1.160 (0.600–2.242)	0.660	0.982 (0.500–1.930)	0.959	0.966 (0.771–1.210)	0.762	0.962 (0.767–1.207)	0.738	
≥ 5	0	1.908 (1.034–3.522)	0.039	1.572 (0.812–3.041)	0.179	1.070 (0.729–1.572)	0.730	1.072 (0.710–1.620)	0.740	
≥ 5	0.5–1	1.268 (0.563–2.854)	0.567	1.185 (0.515–2.727)	0.690	0.930 (0.596–1.450)	0.749	1.034 (0.646–1.654)	0.890	
≥ 5	2–4	1.605 (0.728–3.535)	0.241	1.377 (0.605–3.135)	0.446	0.659 (0.417–1.041)	0.074	0.748 (0.462–1.213)	0.239	
≥ 5	≥ 5	1.884 (0.815–4.354)	0.139	1.568 (0.650–3.783)	0.317	0.646 (0.380–1.096)	0.105	0.596 (0.345–1.030)	0.064	

Multivariable model is adjusted for age, ethnicity, qualification, income, BMI, smoking status, alcohol status, physical activity, diet pattern, HDL, LDL, hypertension and we adjusted for coffee in tea analysis or for tea in coffee analysis

*CI* confidence interval, *HR* hazard ratio, *UK Biobank* United Kingdom Biobank, *AP* angina pectoris, *BMI* body mass index, *HDL* high-density lipoprotein, *LDL* low-density lipoprotein

**Table 4:** Association of coffee and tea with HF in the UK Biobank cohort by sex

Group		Women				Men				P for interaction
		Unadjusted HR (95% CI)	P value	Multi-adjusted HR (95% CI)	P value	Unadjusted HR (95% CI)	P value	Multi-adjusted HR (95% CI)	P value	
Coffee (cups/d)										
0		1.000 (Ref)		1.000 (Ref)		1.000 (Ref)		1.000 (Ref)		0.397
0.5–1		0.825 (0.631–1.078)	0.159	0.847 (0.646–1.112)	0.232	0.876 (0.745–1.030)	0.110	0.913 (0.775–1.075)	0.275	
2–4		0.972 (0.762–1.240)	0.818	0.943 (0.734–1.211)	0.645	0.833 (0.718–0.966)	0.015	0.871 (0.749–1.012)	0.072	
≥ 5		1.029 (0.732–1.446)	0.871	0.858 (0.606–1.215)	0.388	0.971 (0.808–1.168)	0.757	1.043 (0.864–1.258)	0.663	
Tea (cups/d)										
0		1.000 (Ref)		1.000 (Ref)		1.000 (Ref)		1.000 (Ref)		0.581
0.5–1		0.606 (0.424–0.865)	0.006	0.666 (0.466–0.954)	0.027	0.710 (0.568–0.886)	0.003	0.757 (0.605–0.946)	0.014	
2–4		0.725 (0.568–0.926)	0.010	0.739 (0.577–0.946)	0.017	0.900 (0.771–1.050)	0.181	0.874 (0.748–1.023)	0.093	
≥ 5		0.707 (0.540–0.927)	0.012	0.757 (0.576–0.995)	0.046	0.971 (0.825–1.143)	0.723	0.953 (0.809–1.124)	0.571	
Coffee (cups/d)	Tea (cups/d)									
0	0	1.000 (Ref)		1.000 (Ref)		1.000 (Ref)		1.000 (Ref)		0.615
0	0.5–1	0.722 (0.320–1.631)	0.433	0.665 (0.287–1.538)	0.340	0.970 (0.518–1.817)	0.924	0.901 (0.476–1.704)	0.747	
0	2–4	0.698 (0.414–1.179)	0.179	0.609 (0.355–1.047)	0.073	0.989 (0.653–1.498)	0.958	0.889 (0.581–1.361)	0.589	
0	≥ 5	0.690 (0.408–1.164)	0.164	0.608 (0.356–1.037)	0.068	0.953 (0.633–1.434)	0.817	0.885 (0.581–1.349)	0.571	
0.5–1	0	0.833 (0.402–1.729)	0.624	0.638 (0.302–1.346)	0.238	0.941 (0.521–1.702)	0.842	0.959 (0.526–1.749)	0.891	
0.5–1	0.5–1	0.298 (0.112–0.791)	0.015	0.276 (0.103–0.742)	0.011	0.628 (0.354–1.115)	0.113	0.591 (0.330–1.058)	0.077	
0.5–1	2–4	0.600 (0.359–1.004)	0.052	0.467 (0.273–0.799)	0.005	0.912 (0.610–1.363)	0.653	0.764 (0.503–1.159)	0.206	
0.5–1	≥ 5	0.659 (0.388–1.121)	0.124	0.529 (0.304–0.923)	0.025	0.815 (0.537–1.236)	0.335	0.748 (0.486–1.154)	0.189	
2–4	0	1.038 (0.625–1.723)	0.887	0.878 (0.520–1.481)	0.625	1.004 (0.662–1.524)	0.983	1.219 (0.795–1.870)	0.363	
2–4	0.5–1	0.660 (0.366–1.193)	0.169	0.599 (0.327–1.094)	0.096	0.920 (0.701–1.206)	0.544	0.933 (0.710–1.226)	0.620	
2–4	2–4	0.667 (0.406–1.095)	0.110	0.585 (0.348–0.985)	0.044	0.594 (0.423–0.834)	0.003	0.684 (0.487–0.961)	0.029	
2–4	≥ 5	0.494 (0.258–0.946)	0.033	0.440 (0.226–0.858)	0.016	0.776 (0.613–0.983)	0.035	0.803 (0.633–1.019)	0.071	
≥ 5	0	0.714 (0.395–1.294)	0.267	0.556 (0.293–1.054)	0.072	1.014 (0.662–1.555)	0.948	0.868 (0.552–1.366)	0.542	
≥ 5	0.5–1	0.478 (0.193–1.184)	0.111	0.413 (0.165–1.036)	0.060	0.778 (0.468–1.293)	0.334	0.706 (0.416–1.199)	0.198	
≥ 5	2–4	1.021 (0.492–2.119)	0.955	0.670 (0.309–1.453)	0.310	0.894 (0.556–1.440)	0.645	0.895 (0.541–1.482)	0.667	
≥ 5	≥ 5	1.004 (0.426–2.368)	0.992	0.791 (0.325–1.922)	0.604	1.068 (0.635–1.796)	0.803	0.904 (0.526–1.552)	0.714	

Multivariable model is adjusted for age, ethnicity, qualification, income, BMI, smoking status, alcohol status, physical activity, diet pattern, HDL, LDL, hypertension and we adjusted for coffee in tea analysis or for tea in coffee analysis

*CI* confidence interval, *HR* hazard ratio, *UK Biobank* United Kingdom Biobank, *HF* heart failure, *BMI* body mass index, *HDL* high-density lipoprotein, *LDL* low-density lipoprotein

**Table 5:** Association of coffee and tea with stroke in the UK Biobank cohort by smoking status

Group		Smoking status-never				Smoking status-former				Smoking status-current				P for interaction
		Unadjusted HR (95% CI)	P value	Multi-adjusted HR (95% CI)	P value	Unadjusted HR (95% CI)	P value	Multi-adjusted HR (95% CI)	P value	Unadjusted HR (95% CI)	P value	Multi-adjusted HR (95% CI)	P value	
Coffee (cups/d)														
0		1.000 (Ref)		1.000 (Ref)		1.000 (Ref)		1.000 (Ref)		1.000 (Ref)		1.000 (Ref)		0.238
0.5–1		1.788 (0.937–3.413)	0.078	0.709 (0.468–1.073)	0.104	0.584 (0.409–0.833)	0.003	0.648 (0.452–0.927)	0.018	0.872 (0.501–1.519)	0.629	0.667 (0.336–1.326)	0.248	
2–4		1.187 (0.608–2.318)	0.616	0.878 (0.609–1.267)	0.486	0.708 (0.521–0.962)	0.027	0.792 (0.579–1.082)	0.143	0.543 (0.287–1.028)	0.061	0.659 (0.359–1.207)	0.177	
≥ 5		1.493 (0.788–2.828)	0.219	0.597 (0.311–1.147)	0.122	0.633 (0.416–0.963)	0.033	0.776 (0.507–1.187)	0.242	0.526 (0.302–0.917)	0.023	1.216 (0.688–2.149)	0.501	
Tea (cups/d)														
0		1.000 (Ref)		1.000 (Ref)		1.000 (Ref)		1.000 (Ref)		1.000 (Ref)		1.000 (Ref)		0.731
0.5–1		0.877 (0.500–1.538)	0.647	0.869 (0.494–1.530)	0.627	0.816 (0.524–1.272)	0.369	0.814 (0.522–1.270)	0.366	1.422 (0.846–2.391)	0.184	0.621 (0.281–1.369)	0.237	
2–4		0.922 (0.603–1.410)	0.707	0.821 (0.534–1.263)	0.371	0.710 (0.511–0.987)	0.042	0.664 (0.475–0.927)	0.016	0.800 (0.364–1.755)	0.578	0.533 (0.301–0.941)	0.030	
≥ 5		1.115 (0.716–1.737)	0.629	1.050 (0.671–1.642)	0.831	0.868 (0.615–1.226)	0.421	0.814 (0.574–1.155)	0.249	0.729 (0.417–1.275)	0.268	0.629 (0.369–1.070)	0.087	
Coffee (cups/d)	Tea (cups/d)													
0	0	1.000 (Ref)		1.000 (Ref)		1.000 (Ref)		1.000 (Ref)		1.000 (Ref)		1.000 (Ref)		0.547
0	0.5–1	0.776 (0.201–3.003)	0.714	0.596 (0.149–2.394)	0.466	0.994 (0.182–5.430)	0.995	0.588 (0.206–1.675)	0.320	0.485 (0.054–4.365)	0.518	0.504 (0.047–5.429)	0.572	
0	2–4	0.960 (0.410–2.247)	0.925	0.676 (0.282–1.621)	0.380	1.338 (0.461–3.883)	0.592	0.347 (0.81–1.485)	0.154	0.452 (0.113–1.808)	0.261	0.231 (0.043–1.234)	0.086	
0	≥ 5	1.137 (0.490–2.639)	0.765	0.852 (0.361–2.014)	0.715	1.927 (0.689–5.387)	0.211	0.648 (0.381–1.101)	0.109	0.827 (0.270–2.539)	0.740	0.379 (0.102–1.104)	1.146	
0.5–1	0	0.199 (0.024–1.672)	0.137	0.137 (0.014–1.317)	0.085	1.466 (0.393–5.463)	0.569	1.174 (0.212–6.511)	0.854	1.466 (0.393–5.463)	0.569	0.810 (0.570–1.150)	0.236	
0.5–1	0.5–1	0.492 (0.127–1.902)	0.304	0.474 (0.119–1.882)	0.289	0.591 (0.159–2.201)	0.433	0.474 (0.122–1.841)	0.281	0.941 (0.156–5.675)	0.947	3.043 (0.362–25.597)	0.306	
0.5–1	2–4	0.661 (0.276–1.583)	0.352	0.552 (0.219–1.391)	0.208	0.707 (0.275–1.814)	0.470	0.537 (0.200–1.444)	0.218	0.259 (0.043–1.549)	0.139	0.259 (0.040–1.684)	0.157	
0.5–1	≥ 5	0.864 (0.356–2.096)	0.747	0.830 (0.324–2.126)	0.699	0.413 (0.149–1.148)	0.090	0.327 (0.113–0.946)	0.039	0.426 (0.086–2.116)	0.297	0.340 (0.063–1.831)	0.209	
2–4	0	1.198 (0.464–3.091)	0.709	1.321 (0.495–3.522)	0.579	1.411 (0.493–4.042)	0.521	1.363 (0.453–4.098)	0.582	0.754 (0.226–2.521)	0.647	0.427 (0.098–1.857)	0.256	
2–4	0.5–1	1.259 (0.594–2.665)	0.548	1.325 (0.622–2.822)	0.465	0.916 (0.678–1.239)	0.570	0.945 (0.698–1.280)	0.714	0.471 (0.166–1.333)	0.156	0.503 (0.176–1.435)	0.199	
2–4	2–4	1.157 (0.510–2.624)	0.727	1.360 (0.593–3.120)	0.468	0.771 (0.604–0.984)	0.037	0.770 (0.600–0.988)	0.040	0.345 (0.122–0.976)	0.477	0.897 (0.560–1.439)	0.653	
2–4	≥ 5	0.908 (0.447–1.847)	0.790	0.985 (0.482–2.011)	0.966	0.908 (0.681–1.210)	0.511	0.913 (0.680–1.225)	0.544	0.345 (0.122–0.976)	0.045	0.321 (0.113–0.915)	0.033	
≥ 5	0	0.64090.203–2.020)	0.447	0.735 (0.204–2.644)	0.637	1.293 (0.440–3.806)	0.640	1.407 (0.440–4.495)	0.565	0.824 (0.272–2.494)	0.731	0.570 (0.167–1.941)	0.368	
≥ 5	0.5–1	0.460 (0.095–2.219)	0.333	0.513 (0.099–2.664)	0.427	0.954 (0.714–1.274)	0.750	1.009 (0.751–1.357)	0.951	0.867 (0.573–1.313)	0.502	0.842 (0.551–1.286)	0.426	
≥ 5	2–4	0.843 (0.218–3.266)	0.805	0.851 (0.188–3.856)	0.835	0.729 (0.509–1.045)	0.086	0.738 (0.511–1.067)	0.106	0.776 (0.513–1.173)	0.229	0.773 (0.498–1.200)	0.251	
≥ 5	≥ 5	0.334 (0.041–2.722)	0.306	0.356 (0.042–3.014)	0.343	0.745 (0.458–1.211)	0.235	0.694 (0.425–1.134)	0.145	1.258 (0.943–1.680)	0.119	1.200 (0.883–1.629)	0.244	

Multivariable model is adjusted for sex, age, ethnicity, qualification, income, BMI, alcohol status, physical activity, diet pattern, HDL, LDL, hypertension and we adjusted for coffee in tea analysis or for tea in coffee analysis

*CI* confidence interval, *HR* hazard ratio, *UK Biobank* United Kingdom Biobank, *BMI* body mass index, *HDL* high-density lipoprotein, *LDL* low-density lipoprotein

**Table 6:** Association of coffee and tea with AP in the UK Biobank cohort by smoking status

Group		Smoking status-never				Smoking status-former				Smoking status-current				P for interaction
		Unadjusted HR (95% CI)	P value	Multi-adjusted HR (95% CI)	P value	Unadjusted HR (95% CI)	P value	Multi-adjusted HR (95% CI)	P value	Unadjusted HR (95% CI)	P value	Multi-adjusted HR (95% CI)	P value	
Coffee (cups/d)														
0		1.000 (Ref)		1.000 (Ref)		1.000 (Ref)		1.000 (Ref)		1.000 (Ref)		1.000 (Ref)		0.794
0.5–1		1.095 (0.835–1.437)	0.512	0.840 (0.688–1.027)	0.090	0.765 (0.640–0.913)	0.003	0.839 (0.701–1.004)	0.055	0.954 (0.653–1.394)	0.808	1.024 (0.694–1.510)	0.906	
2–4		0.888 (0.673–1.172)	0.402	0.734 (0.605–0.890)	0.002	0.808 (0.688–0.948)	0.009	0.887 (0.754–1.044)	0.149	0.825 (0.583–1.166)	0.276	0.859 (0.602–1.226)	0.403	
≥ 5		0.794 (0.606–1.040)	0.094	0.888 (0.674–1.170)	0.400	0.884 (0.723–1.082)	0.233	1.008 (0.822–1.237)	0.938	0.989 (0.688–1.423)	0.953	1.039 (0.716–1.509)	0.839	
Tea (cups/d)														
0		1.000 (Ref)		1.000 (Ref)		1.000 (Ref)		1.000 (Ref)		1.000 (Ref)		1.000 (Ref)		0.021
0.5–1		1.025 (0.771–1.361)	0.867	1.044 (0.785–1.389)	0.767	0.890 (0.709–1.117)	0.313	0.919 (0.732–1.154)	0.466	1.099 (0.806–1.499)	0.549	0.649 (0.398–1.059)	0.084	
2–4		1.169 (0.940–1.453)	0.161	1.114 (0.894–1.389)	0.337	0.894 (0.756–1.057)	0.189	0.894 (0.754–1.059)	0.193	0.652 (0.405–1.048)	0.077	0.581 (0.411–0.822)	0.002	
≥ 5		1.117 (0.882–1.413)	0.358	1.116 (0.880–1.414)	0.365	0.972 (0.814–1.162)	0.757	0.970 (0.810–1.161)	0.738	0.623 (0.451–0.860)	0.004	0.936 (0.682–1.285)	0.682	
Coffee (cups/d)	Tea (cups/d)													
0	0	1.000 (Ref)		1.000 (Ref)		1.000 (Ref)		1.000 (Ref)		1.000 (Ref)		1.000 (Ref)		0.069
0	0.5–1	1.114 (0.561–2.212)	0.759	0.977 (0.482–1.983)	0.949	0.858 (0.393–1.874)	0.701	0.694 (0.315–1.530)	0.365	1.277 (0.304–5.369)	0.739	1.231 (0.276–5.486)	0.785	
0	2–4	1.464 (0.922–2.326)	0.106	1.220 (0.756–1.966)	0.415	0.932 (0.577–1.504)	0.772	0.837 (0.514–1.361)	0.473	0.838 (0.281–2.502)	0.752	0.692 (0.208–2.307)	0.549	
0	≥ 5	1.233 (0.768–1.981)	0.386	1.123 (0.692–1.823)	0.638	1.087 (0.684–1.727)	0.724	1.036 (0.646–1.663)	0.883	1.987 (0.782–0.051)	0.149	1.813 (0.657–5.002)	0.251	
0.5–1	0	0.676 (0.311–1.469)	0.323	0.570 (0.260–1.248)	0.160	0.816 (0.420–1.582)	0.547	0.917 (0.439–1.918)	0.819	0.739 (0.176–3.096)	0.679	2.415 (0.142–41.184)	0.542	
0.5–1	0.5–1	1.034 (0.564–1.896)	0.914	0.992 (0.535–1.838)	0.979	0.815 (0.407–1.632)	0.563	0.749 (0.369–1.522)	0.425	0.611 (0.123–3.028)	0.546	0.551 (0.102–2.983)	0.489	
0.5–1	2–4	1.157 (0.728–1.839)	0.537	1.005 (0.620–1.629)	0.985	1.000 (0.586–1.706)	1.000	0.873 (0.502–1.518)	0.630	0.552 (0.154–1.982)	0.362	0.515 (0.134–1.974)	0.333	
0.5–1	≥ 5	0.947 (0.578–1.554)	0.831	0.570 (0.260–1.248)	0.578	0.872 (0.504–1.509)	0.624	0.770 (0.437–1.358)	0.366	1.838 (0.564–5.996)	0.313	2.907 (0.845–9.996)	0.090	
2–4	0	1.024 (0.610–1.720)	0.928	1.250 (0.732–2.137)	0.414	0.832 (0.515–1.343)	0.452	0.835 (0.509–1.369)	0.474	1.464 (0.549–3.903)	0.446	1.011(0.331–3.086)	0.985	
2–4	0.5–1	0.984 (0.653–1.481)	0.937	1.039 (0.688–1.568)	0.856	0.941 (0.797–1.113)	0.479	0.985 (0.833–1.165)	0.861	0.917 (0.618–1.359)	0.665	0.953 (0.641–1.417)	0.811	
2–4	2–4	0.909 (0.581–1.422)	0.676	0.993 (0.632–1.560)	0.976	0.962 (0.847–1.093)	0.554	0.990 (0.870–1.127)	0.884	0.937 (0.712–1.234)	0.645	0.965 (0.729–1.277)	0.803	
2–4	≥ 5	0.910 (0.631–1.313)	0.616	0.953 (0.659–1.377)	0.798	1.031 (0.885–1.200)	0.697	1.078 (0.923–1.259)	0.344	0.774 (0.541–1.107)	0.161	0.750 (0.523–1.075)	0.117	
≥ 5	0	1.104 (0.628–1.938)	0.731	0.963 (0.525–1.769)	0.904	0.967 (0.603–1.582)	0.922	1.048 (0.636–1.727)	0.855	2.216 (0.878–5.596)	0.092	2.042 (0.772–5.402)	0.151	
≥ 5	0.5–1	1.086 (0.563–2.095)	0.806	0.959 (0.479–1.921)	0.906	1.018 (0.891–1.163)	0.795	1.038 (0.907–1.188)	0.589	0.777 (0.583–1.035)	0.084	0.843 (0.629–1.130)	0.253	
≥ 5	2–4	1.212 (0.620–2.370)	0.573	0.992 (0.481–2.046)	0.982	0.890 (0.777–1.030)	0.121	0.945 (0.819–1.089)	0.434	0.699 (0.536–0.912)	0.008	0.744 (0.563–0.985)	0.039	
≥ 5	≥ 5	1.387 (0.698–2.753)	0.350	1.138 (0.558–2.322)	0.722	0.888 (0.734–1.073)	0.218	0.842 (0.696–1.020)	0.078	0.718 (0.527–0.978)	0.036	0.682 (0.495–0.938)	0.018	

Multivariable model is adjusted for sex, age, ethnicity, qualification, income, BMI, alcohol status, physical activity, diet pattern, HDL, LDL, hypertension and we adjusted for coffee in tea analysis or for tea in coffee analysis

*CI* confidence interval, *HR* hazard ratio, *UK Biobank* United Kingdom Biobank, *AP* angina pectoris, *BMI* body mass index, *HDL* high-density lipoprotein, *LDL* low-density lipoprotein

**Table 7:** Association of coffee and tea with HF in the UK Biobank cohort by smoking status

Group		Smoking status-never				Smoking status-former				Smoking status-current				P for interaction
		Unadjusted HR (95% CI)	P value	Multi-adjusted HR (95% CI)	P value	Unadjusted HR (95% CI)	P value	Multi-adjusted HR (95% CI)	P value	Unadjusted HR (95% CI)	P value	Multi-adjusted HR (95% CI)	P value	
Coffee (cups/d)														
0		1.000 (Ref)		1.000 (Ref)		1.000 (Ref)		1.000 (Ref)		1.000 (Ref)		1.000 (Ref)		0.667
0.5–1		1.190 (0.865–1.637)	0.284	0.958 (0.768–1.195)	0.702	0.822 (0.671–1.005)	0.056	0.887 (0.724–1.087)	0.249	0.837 (0.560–1.251)	0.384	0.793 (0.526–1.197)	0.270	
2–4		1.082 (0.786–1.491)	0.629	0.794 (0.641–0.984)	0.035	0.919 (0.767–1.102)	0.362	0.995 (0.828–1.195)	0.956	0.882 (0.620–1.255)	0.486	0.834 (0.580–1.198)	0.325	
≥ 5		0.936 (0.683–1.282)	0.681	0.824 (0.597–1.138)	0.241	0.976 (0.778–1.225)	0.836	1.109 (0.881–1.395)	0.380	0.999 (0.688–1.450)	0.994	0.995 (0.680–1.455)	0.978	
Tea (cups/d)														
0		1.000 (Ref)		1.000 (Ref)		1.000 (Ref)		1.000 (Ref)		1.000 (Ref)		1.000 (Ref)		0.418
0.5–1		0.838 (0.613–1.146)	0.269	0.858 (0.627–1.174)	0.338	0.673 (0.515–0.878)	0.004	0.701 (0.537–0.916)	0.009	1.056 (0.763–1.461)	0.744	0.528 (0.304–0.915)	0.023	
2–4		0.998 (0.792–1.258)	0.988	0.923(0.730–1.166)	0.500	0.852 (0.711–1.021)	0.083	0.838 (0.698–1.006)	0.058	0.527 (0.308–0.900)	0.019	0.666 (0.469–0.945)	0.023	
≥ 5		0.947 (0.737–1.218)	0.673	0.908 (0.704–1.169)	0.453	0.889 (0.732–1.080)	0.235	0.899 (0.738–1.094)	0.288	0.736 (0.533–1.016)	0.062	0.908 (0.652–1.266)	0.570	
Coffee (cups/d)	Tea (cups/d)													
0	0	1.000 (Ref)		1.000 (Ref)		1.000 (Ref)		1.000 (Ref)		1.000 (Ref)		1.000 (Ref)		0.771
0	0.5–1	1.015 (0.502–2.052)	0.967	0.991(0.477–2.060)	0.981	0.957 (0.448–2.046)	0.910	0.898 (0.825–0.978)	0.013	0.288 (0.035–2.347)	0.245	0.132 (0.015–1.153)	0.067	
0	2–4	1.050 (0.652–1.692)	0.840	0.885(0.541–1.449)	0.627	0.751 (0.454–1.242)	0.264	0.951 (0.895–1.011)	0.106	0.848 (0.342–2.103)	0.722	0.326 (0.111–0.964)	0.043	
0	≥ 5	0.875 (0.536–1.429)	0.594	0.736 (0.445–1.219)	0.234	0.768 (0.472–1.252)	0.290	1.003 (0.931–1.082)	0.931	1.029 (0.452–2.345)	0.945	0.495 (0.195–1.259)	0.140	
0.5–1	0	0.947 (0.469–1.914)	0.880	0.832 (0.407–1.701)	0.614	0.803 (0.405–1.593)	0.530	0.973 (0.441–2.145)	0.946	1.152 (0.296–4.483)	0.839	1.222 (0.101–4.714)	0.875	
0.5–1	0.5–1	0.776 (0.402–1.496)	0.449	0.769 (0.395–1.497)	0.440	0.410 (0.172–0.977)	0.044	0.314 (0.130–0.759)	0.010	0.414 (0.069–2.483)	0.335	0.468 (0.074–2.972)	0.420	
0.5–1	2–4	0.957 (0.598–1.532)	0.854	0.780 (0.477–1.276)	0.323	0.865 (0.496–1.058)	0.608	0.630 (0.354–1.122)	0.117	0.790 (0.227–2.755)	0.712	0.642 (1.176–2.344)	0.503	
0.5–1	≥ 5	0.823 (0.498–1.362)	0.449	0.732 (0.433–1.236)	0.243	0.756 (0.426–1.342)	0.339	0.611 (0.337–1.106)	0.104	1.225 (0.367–4.087)	0.741	1.020 (0.293–3.543)	0.976	
2–4	0	1.201 (0.704–2.048)	0.501	1.451 (0.837–2.516)	0.185	0.811 (0.496–1.326)	0.404	0.741 (0.444–1.239)	0.254	1.109 (0.473–2.598)	0.812	0.568 (0.200–1.615)	0.289	
2–4	0.5–1	1.206 (0.788–1.846)	0.388	1.278 (0.832–1.963)	0.262	0.820 (0.677–0.993)	0.042	0.874 (0.721–1.060)	0.171	0.886 (0.601–1.307)	0.542	0.946 (0.637–1.403)	0.781	
2–4	2–4	0.719 (0.428–1.209)	0.214	0.802 (0.475–1.354)	0.409	0.931 (0.814–1.066)	0.302	0.957 (0.835–1.098)	0.531	0.803 (0.607–1.062)	0.124	0.790 (0.595–1.050)	0.105	
2–4	≥ 5	0.833 (0.557–1.247)	0.374	0.888 (0.592–1.331)	0.564	1.031 (0.879–1.211)	0.705	1.045 (0.888–1.230)	0.597	0.853 (0.617–1.177)	0.333	0.828 (0.598–1.146)	0.256	
≥ 5	0	0.559 (0.286–1.095)	0.090	0.505 (0.247–1.029)	0.060	0.828 (0.499–1.372)	0.464	0.840 (0.497–1.421)	0.516	1.024 (0.452–2.321)	0.954	0.699 (0.291–1.679)	0.423	
≥ 5	0.5–1	0.920 (0.462–1.830)	0.811	0.876 (0.426–1.804)	0.720	0.918 (0.778–1.084)	0.312	0.925 (0.782–1.094)	0.364	0.745 (0.527–1.055)	0.098	0.774 (0.544–1.101)	0.154	
≥ 5	2–4	1.303 (0.666–2.550)	0.440	1.382 (0.661–2.889)	0.389	0.962 (0.832–1.114)	0.607	1.024 (0.883–1.187)	0.757	0.903 (0.713–1.143)	0.397	0.919 (0.715–1.182)	0.512	
≥ 5	≥ 5	0.840 (0.373–1.890)	0.673	0.784 (0.336–1.832)	0.575	1.021 (0.849–1.226)	0.828	0.969 (0.806–1.166)	0.741	1.124 (0.900–1.403)	0.302	1.162 (0.911–1.481)	0.226	

Multivariable model is adjusted for sex, age, ethnicity, qualification, income, BMI, alcohol status, physical activity, diet pattern, HDL, LDL, hypertension and we adjusted for coffee in tea analysis or for tea in coffee analysis

*CI* confidence interval, *HR* hazard ratio, *UK Biobank* United Kingdom Biobank, *HF* heart failure, *BMI* body mass index, *HDL* high-density lipoprotein, *LDL* low-density lipoprotein

## Discussion

In this large prospective cohort study, we found that among patients with T2DM: (1) the consumption of 2–4 cups of tea alone or 0.5–1 cup of coffee per day was found to be associated with a reduced risk of stroke. (2) the consumption of 2–4 cups of coffee alone per day was associated with a reduced risk of AP. (3) the consumption of 0.5–1 cup of tea alone per day or a combination of 2–4 cups of coffee and 0.5–1 cup of tea per day was associated with a reduced risk of HF.

An Inverse relationship of coffee consumption with risk of macrovascular complications has been widely reported in previous prospective studies conducted in the general population [38]. Only a few studies have been focused on the coffee–macrovascular complications association among individuals with T2DM [29, 30]. Previous studies have investigated the relationship between the individual consumption of coffee and tea and the risk of stroke in both general populations [39–42] and patients with T2DM [10, 13], but the results have been inconsistent. Our study revealed nonlinear associations between coffee/tea intake and macrovascular complications in T2DM. The nonlinearity may reflect a balance between protective and adverse components. For coffee, caffeine improve endothelial function at moderate doses but may induce vascular stiffness at high doses [23]. For tea, polyphenols exhibit antioxidant effects that plateau beyond a certain intake [43]. In addition, our findings support the association between the consumption of tea and coffee and a reduced risk of stroke, which aligns with a review that summarized existing evidence from experimental studies, prospective studies, and meta-analyses, reporting that the consumption of tea and coffee may be associated with a reduced risk of stroke [44]. One possible mechanism for this relationship is that coffee and tea are negatively associated with endothelial dysfunction, which is a major cause of stroke [45–48]. Another potential mechanism could be that coffee contains caffeine and is a rich source of antioxidants, with evidence suggesting that coffee is negatively associated with cardiometabolic risks, including macrovascular complications, T2DM, blood lipids, and hypertension [49]. Similarly, tea consumption may reduce stroke risk through multiple pathways. First, tea polyphenols (such as catechins in green tea and theaflavins in black tea) can enhance the bioavailability of nitric oxide, reduce oxidative stress, and thereby improve endothelial function [43, 48]. Second, flavonoids in tea can inhibit pro-inflammatory cytokines associated with atherosclerosis (such as IL-6 and TNF-α) [50]. Third, tea components can improve insulin sensitivity [27] and lipid profiles [3], thereby indirectly reducing the risk of stroke in T2DM patients. While these explanations are biologically plausible, further research is needed to elucidate the exact underlying mechanisms of coffee and tea consumption in the occurrence of stroke. Furthermore, our findings are not consistent with previous studies that have reported a negative correlation between tea consumption and AP [51], as well as an association between coffee intake and HF event rates [52]. These discrepancies may be attributed to variations in sample size, study design, ethnic background, and the classification of coffee and tea consumption.

Our research indicates that there is an interaction between coffee and tea that is associated with HF. Several mechanisms may explain the potential link between the combination of coffee and tea and HF. Firstly, coffee is a primary source of caffeine and contains phenolic compounds and other bioactive substances with potential health benefits. Similarly, tea contains caffeine, and flavonoids, which have been reported to exhibit antioxidative stress and anti-inflammatory effects [43]. Coffee and tea are two distinct beverages that share and diverge in numerous components [53]. One potential mechanism could be the combined protective effect of the ingredients contained in both beverages [54]. Secondly, it is vital to acknowledge the presence of specific polyphenols in coffee and tea. Coffee is rich in hydroxycinnamic acid, while tea is dominated by catechins. These polyphenols have been proven to improve endothelial function, insulin resistance, and anti-inflammatory activity [55]. Therefore, the specific polyphenol contents of coffee and tea may play a combined protective role in the pathogenesis of HF. Thirdly, the interaction between coffee and tea in relation to HF may be coincidental. Finally, coffee and tea consumption may co-regulate the activation of certain cytokines [50, 56, 57]. Notably, our stratified analyses revealed differential associations across subgroups. When stratified by smoking status, we observed a more pronounced inverse association between moderate tea consumption (2–4 cups/day) and reduced risk of angina pectoris (AP) among patients with T2DM (P-interaction = 0.021). However, this effect modification was not evident for stroke or heart failure outcomes. In contrast, the beneficial associations between coffee consumption and risk reduction for stroke, AP, and heart failure remained consistent across all subgroups, showing no significant effect modification by sex (all P-interaction > 0.05), smoking status, physical activity level, or BMI categories. This pattern of non-differential effects for coffee consumption aligns with previous epidemiological reports [58], suggesting that coffee’s cardioprotective mechanisms may operate independently of these demographic and lifestyle factors.

The merits of this study lie in the substantial sample size of UK Biobank participants and the prospective design. Our current study also has some limitations. Firstly, it should be noted that the initial data collection procedure involved the subjects providing self-reported information regarding their habitual consumption of coffee and tea. This method of data collection may not have reflected long-term consumption patterns. It is imperative that future studies investigate the impact of changes in coffee and tea intake over time on cardiovascular risk. Secondly, it should be noted that both coffee and tea intake are self-reported indicators, which may lead to inaccurate responses. However, it is important to acknowledge that most large epidemiological studies rely on self-reported questionnaires. Thirdly, evidence suggests that volunteers in the UK Biobank cohort tend to be more health-conscious than non-participants, resulting in a “volunteer bias” [59]. Thus, conclusions may be influenced by lower absolute risk and possible residual confounders.

Macrovascular complications are the primary cause of mortality in individuals with T2DM, making prevention of macrovascular complications particularly crucial for this population. Despite advancements in understanding the pathophysiology of stroke, AP, and HF, their clinical management remains unsatisfactory. Therefore, it is essential to identify modifiable risk factors for stroke, AP, and HF. Our findings suggest a potential beneficial association between moderate coffee and tea consumption and the risk of stroke, AP, and HF; however, this study cannot establish causation. Lifestyle interventions such as promoting healthy dietary habits (e.g., moderate coffee and tea consumption) may benefit the T2DM population by reducing the risk of stroke, AP, and HF. From a public health perspective, given that regular tea and coffee drinkers make up a significant portion of the population, even if the potential health benefits or risks associated with tea and coffee intake are small, they could have important public health implications.

## Conclusions

In conclusion, our study suggests that, after adjusting for potential confounding factors, moderate consumption of coffee and tea is associated with a reduced risk of stroke and moderate coffee consumption is associated with a reduced risk of AP in patients with T2DM. Furthermore, drinking tea alone or with coffee was associated with a reduced risk of HF.

## Supplementary Information

Supplementary Material 1.

## Data Availability

The data underlying this article are available in UK Biobank at https://www.ukbiobank.ac.uk/. This research has been conducted using the UK Biobank Resource under Application Number 98124. The datasets analyzed during the current study are available from the corresponding author on reasonable request.

## Abbreviations

HR: Hazard ratio
CI: Confidence interval
AP: Angina pectoris
HF: Heart failure
T2DM: Type 2 diabetes mellitus
BMI: Body mass index
HDL: High-density lipoprotein
LDL: Low-density lipoprotein
SD: Standard deviation
