# Supplementary material for: Association between coffee and tea consumption and the risk of macrovascular complications in type 2 diabetes: a UK Biobank cohort study
Source: Diabetol Metab Syndr. 2025 Jun 19;17:233. doi: 10.1186/s13098-025-01807-4 (PMC12178019; doi:10.1186/s13098-025-01807-4)
Supplement: Supplementary file 1 — Supplementary Material 1. [file 13098_2025_1807_MOESM1_ESM.docx]

**Table S1.** Definitions of macrovascular complications and the associated data source.

| macrovascular complications | ICD-10 |
| --- | --- |
| stroke | I64 |
| AP | I20 |
| HF | I50 |

**Table S2.** Diet component definitions used in the UK Biobank study

| Source and definition of healthy diet | Self-reported UK Biobank field code |
| --- | --- |
| UK Biobank Food Frequency  Questionnaire at baseline;  1. Fruits: ≥ 3 servings/day  2. Vegetables: ≥ 3 servings/day  3. Fish: ≥2 servings/week  4. Processed meats: ≤1 serving/week  5. Unprocessed red meats: ≤ 1.5 servings/week  6. Whole grains: ≥ 3servings/day  7. Refined grains: ≤1.5 servings/day | 1309, 1319, 1289, 1299, 1329, 1339, 1349, 1369, 1379, 1389, 1438, 1448, 1458, 1468 |

**Table S3.** Association of coffee and tea with stroke, AP and HF in the UK Biobank cohort (unadjusted models)

| Groups | | Stroke | |  | AP | |  | HF | |
| --- | --- | --- | --- | --- | --- | --- | --- | --- | --- |
|  |  | HR (95% CI) | *P* value |  | HR (95% CI) | *P* value |  | HR (95% CI) | *P* value |
| Coffee (cups/d) | | | | | | | | | |
| 0 | | 1.000 (Ref) |  |  | 1.000 (Ref) |  |  | 1.000 (Ref) |  |
| 0.5-1 | | 0.630 (0.491-0.808) | <0.001 |  | 0.818 (0.722-0.927) | 0.002 |  | 0.871 (0.758-1.000) | 0.051 |
| 2-4 | | 0.764 (0.616-0.948) | 0.014 |  | 0.811 (0.723-0.909) | <0.001 |  | 0.899 (0.792-1.020) | 0.099 |
| ≥5 | | 0.855 (0.644-1.33) | 0.275 |  | 0.983 (0.850-1.136) | 0.815 |  | 1.040 (0.885-1.222) | 0.634 |
| Tea (cups/d) | | | | | | | | | |
| 0 | | 1.000 (Ref) |  |  | 1.000 (Ref) |  |  | 1.00 (Ref) |  |
| 0.5-1 | | 0.744 (0.543-1.020) | 0.066 |  | 0.862 (0.731-1.017) | 0.078 |  | 0.682 (0.565-0.823) | <0.001 |
| 2-4 | | 0.702 (0.556-0.885) | 0.003 |  | 0.918 (0.813-1.037) | 0.170 |  | 0.860 (0.755-0.979) | 0.023 |
| ≥5 | | 0.886 (0.697-1.126) | 0.323 |  | 0.997 (0.877-1.135) | 0.967 |  | 0.908 (0.790-1.044) | 0.175 |
| Coffee (cups/d) | Tea (cups/d) |  |  |  |  |  |  |  |  |
| 0 | 0 | 1.000 (Ref) |  |  | 1.000 (Ref) |  |  | 1.000 (Ref) |  |
| 0 | 0.5-1 | 0.755 (0.293-1.946) | 0.561 |  | 1.011 (0.623-1.639) | 0.965 |  | 0.876 (0.534-1.438) | 0.601 |
| 0 | 2-4 | 0.944 (0.529-1.686) | 0.846 |  | 1.198 (0.873-1.645) | 0.262 |  | 0.906 (0.656-1.250) | 0.547 |
| 0 | ≥5 | 1.345 (0.774-2.339) | 0.293 |  | 1.327 (0.974-1.810) | 0.073 |  | 0.907 (0.661-1.246) | 0.547 |
| 0.5-1 | 0 | 0.780 (0.330-1.846) | 0.573 |  | 0.846 (0.528-1.356) | 0.487 |  | 0.905 (0.572-1.433) | 0.671 |
| 0.5-1 | 0.5-1 | 0.694 (0.312-1.546) | 0.372 |  | 0.865 (0.568-1.316) | 0.498 |  | 0.572 (0.325-0.853) | 0.009 |
| 0.5-1 | 2-4 | 0.700 (0.394-1.242) | 0.222 |  | 1.035 (0.759-1.410) | 0.829 |  | 0.844 (0.618-1.153) | 0.287 |
| 0.5-1 | ≥5 | 0.657 (0.360-1.197) | 0.170 |  | 1.016 (0.738-1.398) | 0.924 |  | 0.793 (0.573-1.097) | 0.161 |
| 2-4 | 0 | 1.132 (0.639-2.007) | 0.670 |  | 1.037 (0.751-1.432) | 0.825 |  | 0.984 (0.714-1.356) | 0.921 |
| 2-4 | 0.5-1 | 0.879 (0.473-1.634) | 0.684 |  | 0.919 (0.652-1.295) | 0.630 |  | 0.633 (0.441-0.910) | 0.014 |
| 2-4 | 2-4 | 0.743 (0.427-1.294) | 0.294 |  | 0.972 (0.718-1.317) | 0.857 |  | 0.778 (0.573-1.055) | 0.107 |
| 2-4 | ≥5 | 0.793 (0.429-1.464) | 0.458 |  | 1.030 (0.740-1.432) | 0.863 |  | 0.926 (0.664-1.290) | 0.649 |
| ≥5 | 0 | 1.155 (0.637-2.096) | 0.635 |  | 1.397 (1.009-1.934) | 0.044 |  | 0.971 (0.693-1.361) | 0.863 |
| ≥5 | 0.5-1 | 0.805 (0.377-1.720) | 0.575 |  | 1.166 (0.793-1.714) | 0.436 |  | 0.748 (0.489-1.143) | 0.179 |
| ≥5 | 2-4 | 0.594 (0.267-1.323) | 0.203 |  | 0.943 (0.636-1.399) | 0.772 |  | 0.986 (0.669-1.451) | 0.942 |
| ≥5 | ≥5 | 1.099 (0.505-2.393) | 0.812 |  | 0.976 (0.624-1.527) | 0.916 |  | 1.122 (0.729-1.725) | 0.601 |

Abbreviations:AP, angina pectoris; HF, heart failure; CI, confidence interval; HR, hazard ratio; UK Biobank, United Kingdom Biobank.

**Table S4.** Risk of developing macrovascular complications according to coffee type in the UK Biobank

| Coffee types | stroke | |  | AP | |  | HF | |
| --- | --- | --- | --- | --- | --- | --- | --- | --- |
|  | HR (95% CI) | P value |  | HR (95% CI) | P value |  | HR (95% CI) | P value |
| Ground coffee vs  instant coffee | 1.294(0.989-1.693) | 0.060 |  | 0.961(0.831-1.111) | 0.592 |  | 1.026 (0.879-1.198) | 0.743 |
| Instant coffee  vs decaffeinated coffee | 1.086(0.811-1.454) | 0.582 |  | 0.871(0.763-0.994) | 0.041 |  | 1.019 (0.876-1.185) | 0.806 |
| Ground coffee  vs decaffeinated coffee | 0.700(0.491-0.999) | 0.049 |  | 1.173(0.984-1.398) | 0.074 |  | 0.939 (0.774-1.141) | 0.528 |

Multivariable model is adjusted for age, ethnicity, qualification, income, BMI, smoking status, alcohol status, physical activity, diet pattern , HDL, LDL, hypertension and tea intake. Abbreviations: AP, angina pectoris; HF, heart failure; CI, confidence interval; HR, hazard ratio; UK Biobank, United Kingdom Biobank; BMI, body mass index; HDL, high-density lipoprotein; LDL, low-density lipoprotein.

**Table S5.** Effect of coffee versus tea consumption on incident macrovascular disease in patients with type 2 diabetes mellitus, with exclusion of cases occurring within the first two years of follow-up

| Groups | | stroke | AP | HF |
| --- | --- | --- | --- | --- |
| Coffee (cups/d) | | | | |
| 0 |  | 1.000 (Ref) | 1.000 (Ref) | 1.000 (Ref) |
| 0.5-1 |  | 0.683(0.525-0.890) | 0.862(0.754-0.986) | 0.880(0.761-1.017) |
| 2-4 |  | 0.810(0.642-1.020) | 0.808(0.714-0.915) | 0.880(0.771-1.006) |
| ≥5 |  | 0.832(0.612-1.130) | 0.980(0.839-1.145) | 0.969(0.817-1.149) |
| P-value for trend |  | 0.043 | 0.003 | 0.183 |
| Tea (cups/d) | | | | |
| 0 |  | 1.000 (Ref) | 1.000 (Ref) | 1.000 (Ref) |
| 0.5-1 |  | 0.754(0.540-1.054) | 0.909(0.761-1.085) | 0.706(0.579-0.862) |
| 2-4 |  | 0.629(0.492-0.805) | 0.907(0.795-1.034) | 0.836(0.729-0.959) |
| ≥5 |  | 0.822(0.639-1.059) | 1.002(0.873-1.151) | 0.901(0.779-1.042 |
| P-value for trend | | 0.003 | 0.244 | 0.004 |
| Coffee (cups/d) | Tea (cups/d) |  |  |  |
| 0 | 0 | 1.000 (Ref) | 1.000 (Ref) | 1.000 (Ref) |
| 0 | 0.5-1 | 0.479(0.173-1.326) | 0.860(0.520-1.422) | 0.713(0.427-1.192) |
| 0 | 2-4 | 0.607(0.335-1.102) | 0.892(0.641-1.241) | 0.682(0.491-0.946) |
| 0 | ≥5 | 0.956(0.544-1.678) | 1.066(0.773-1.470) | 0.701(0.508-0.968) |
| 0.5-1 | 0 | 0.7430(0.314-1.757) | 0.716(0.433-1.182) | 0.762(0.473-1.227) |
| 0.5-1 | 0.5-1 | 0.613(0.267-1.407) | 0.735(0.467-1.156) | 0.429(0.257-0.717) |
| 0.5-1 | 2-4 | 0.516(0.287-0.930) | 0.830(0.601-1.146) | 0.647(0.471-0.889) |
| 0.5-1 | ≥5 | 0.526(0.285-0.970) | 0.917(0.659-1.276) | 0.641(0.461-0.893) |
| 2-4 | 0 | 0.905(0.506-1.619) | 0.792(0.564-1.110) |  |
| 2-4 | 0.5-1 | 0.728(0.385-1.378) | 0.815(0.569-1.165) | 0.747(0.538-1.036) |
| 2-4 | 2-4 | 0.526(0.298-0.929) | 0.805(0.587-1.104) | 0.533(0.368-0.774) |
| 2-4 | ≥5 | 0.552(0.294-1.037) | 0.766(0.541-1.085) | 0.585(0.428-0.799) |
| ≥5 | 0 | 0.754(0.405-1.401) | 1.116(0.796-1.565) | 0.704(0.502-0.987) |
| ≥5 | 0.5-1 | 0.574(0.257-1.284) | 0.945(0.629-1.420) | 0.556(0.357-0.867) |
| ≥5 | 2-4 | 0.463(0.207-1.036) | 0.816(0.543-1.227) | 0.782(0.528-1.159) |
| ≥5 | ≥5 | 0.766(0.343-1.714) | 0.816(0.543-1.227) | 0.760(0.483-1.194) |
| P-value for trend | | 0.016 | 0.034 | 0.041 |

Abbreviations:AP, angina pectoris; HF, heart failure; Multivariable model is adjusted for age, ethnicity, qualification, income, BMI, smoking status, alcohol status, physical activity, diet pattern , HDL, LDL, hypertension and we adjusted for coffee in tea analysis or for tea in coffee analysis. Abbreviations: CI, confidence interval; HR, hazard ratio; UK Biobank, United Kingdom Biobank; BMI, body mass index; HDL, high-density lipoprotein; LDL, low-density lipoprotein.


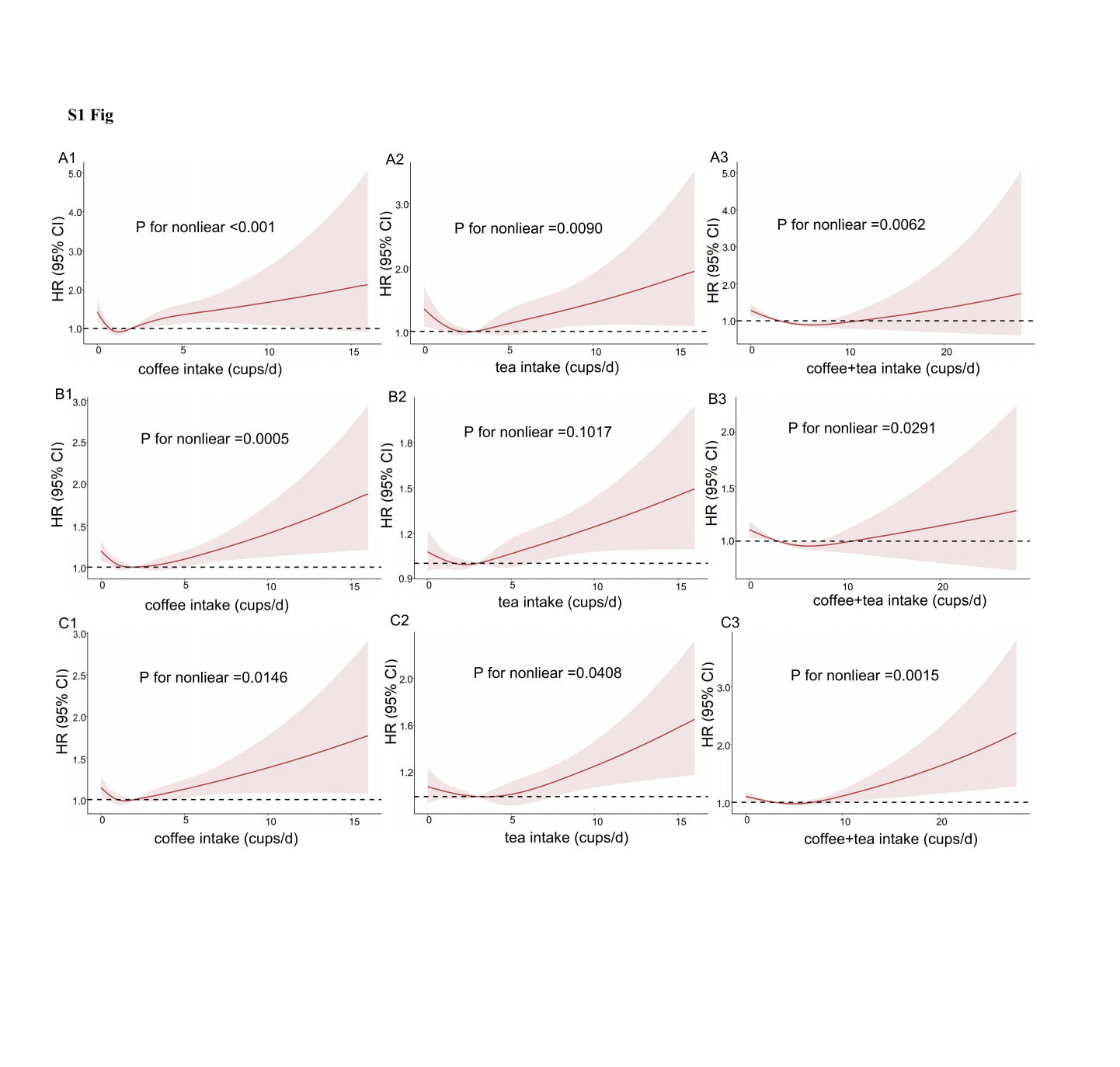


**Figure S1 .** Unadjusted Restricted Cubic Spline models for the relationship between coffee, tea, and their combination with stroke, AP, and HF. (A1) Coffee and stroke; (A2) Tea and stroke; (A3) Combination of coffee and tea on stroke; (B1) Coffee and AP; (B2) Tea and AP; (B3) Combination of coffee and tea on AP; (C1) Coffee and HF; (C2) Tea and HF; (C3) Combination of coffee and tea on HF. The 95% CIs of the adjusted HR are represented by the shaded area. AP, angina pectoris; HF, heart failure.

**
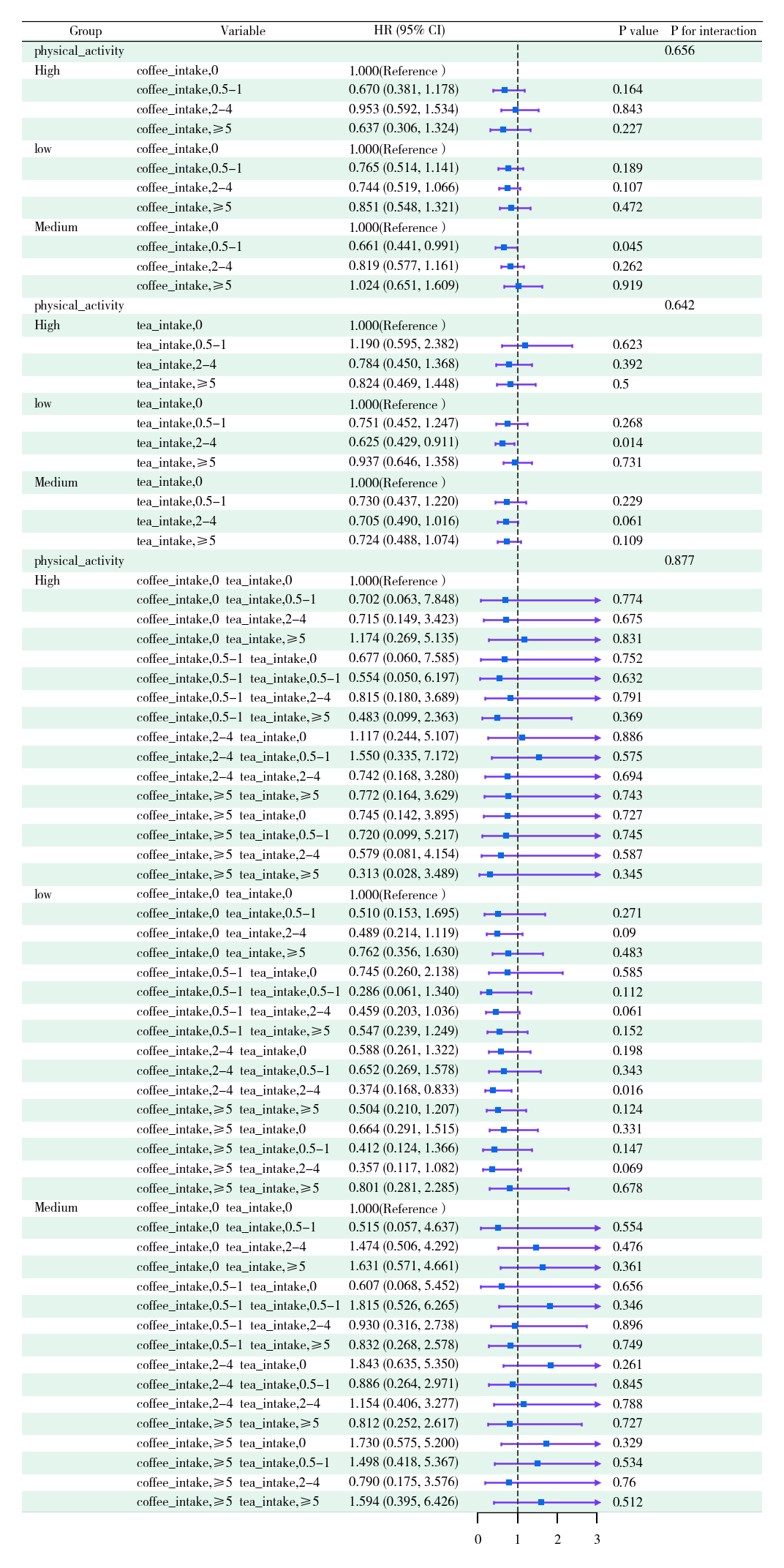
**

**Figure S2 .** Association of coffee and tea with stroke in the UK Biobank cohort by physical activity. Multivariable model is adjusted for age, sex, ethnicity, qualification, income, BMI, smoking status, alcohol status, diet pattern , HDL, LDL, hypertension and we adjusted for coffee in tea analysis or for tea in coffee analysis. Abbreviations: CI, confidence interval; HR, hazard ratio; UK Biobank, United Kingdom Biobank; BMI, body mass index; HDL, high-density lipoprotein; LDL, low-density lipoprotein.


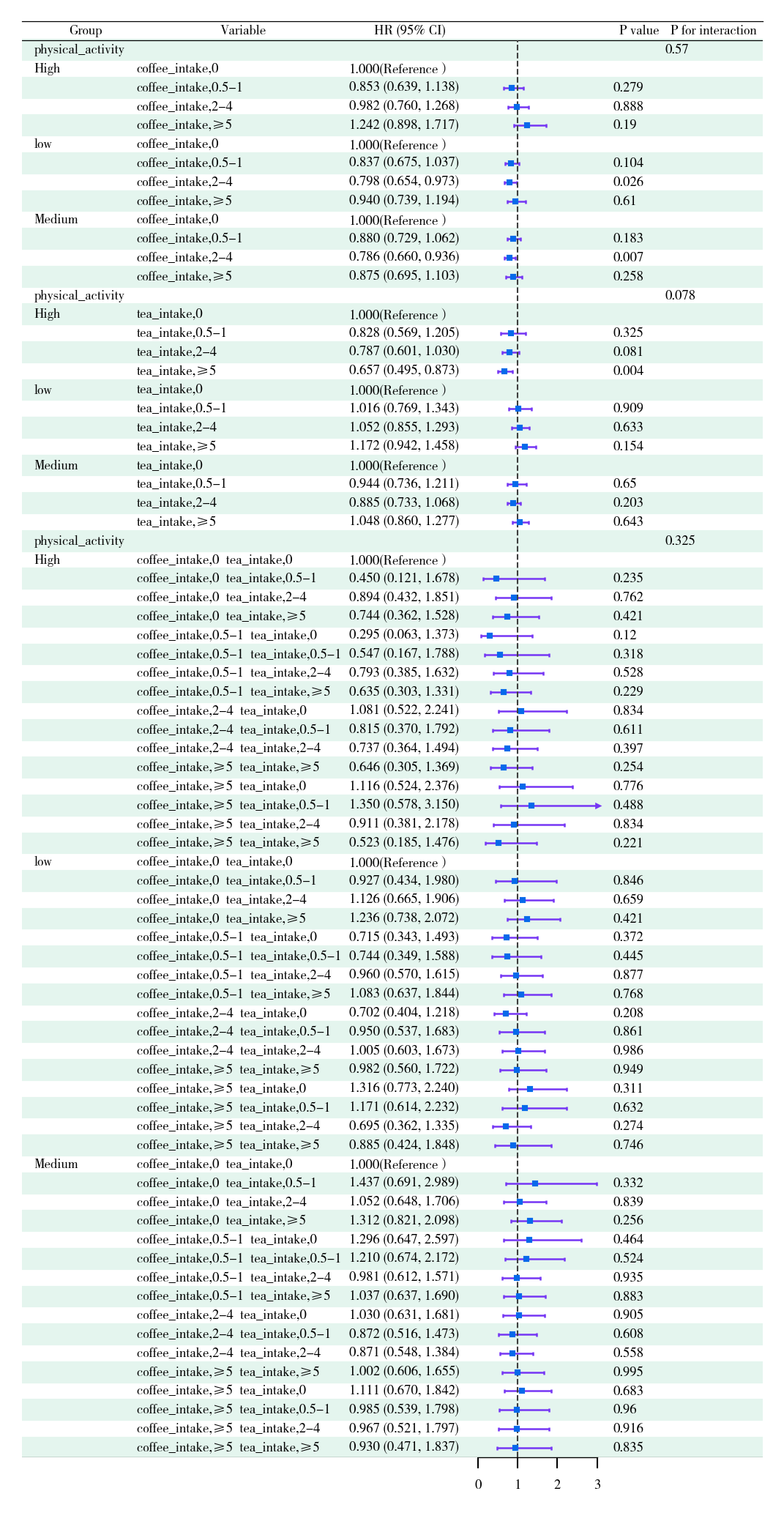


**Figure S3 .** Association of coffee and tea with AP in the UK Biobank cohort by Physical activity. Multivariable model is adjusted for age, sex, ethnicity, qualification, income, BMI, smoking status, alcohol status, diet pattern , HDL, LDL, hypertension and we adjusted for coffee in tea analysis or for tea in coffee analysis. Abbreviations: CI, confidence interval; HR, hazard ratio; UK Biobank, United Kingdom Biobank; BMI, body mass index; HDL, high-density lipoprotein; LDL, low-density lipoprotein.

**
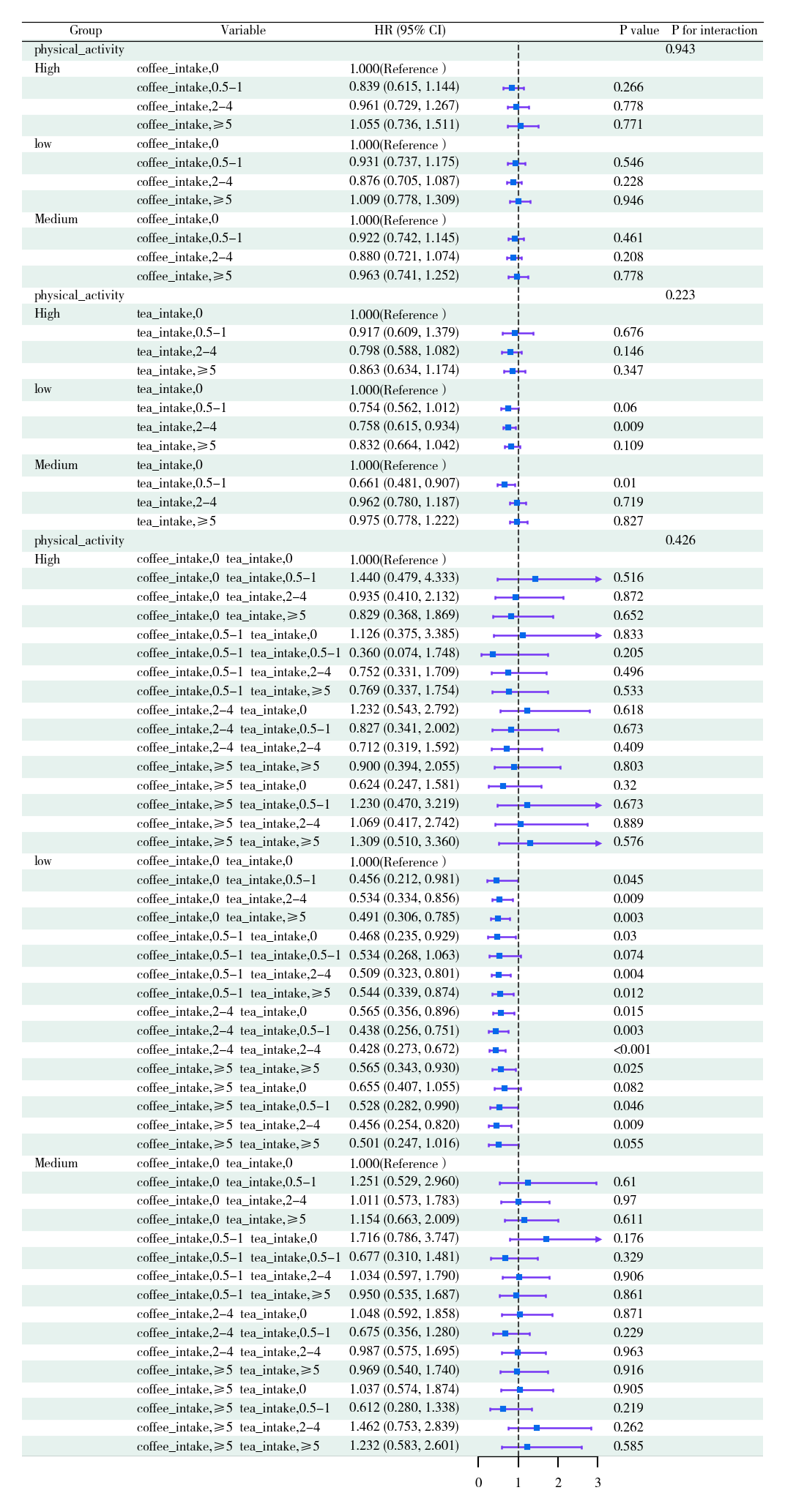
**

**Figure S4 .** Association of coffee and tea with HF in the UK Biobank cohort by Physical activity. Multivariable model is adjusted for age, sex, ethnicity, qualification, income, BMI, smoking status, alcohol status, diet pattern , HDL, LDL, hypertension and we adjusted for coffee in tea analysis or for tea in coffee analysis. Abbreviations: CI, confidence interval; HR, hazard ratio; UK Biobank, United Kingdom Biobank; BMI, body mass index; HDL, high-density lipoprotein; LDL, low-density lipoprotein.

**
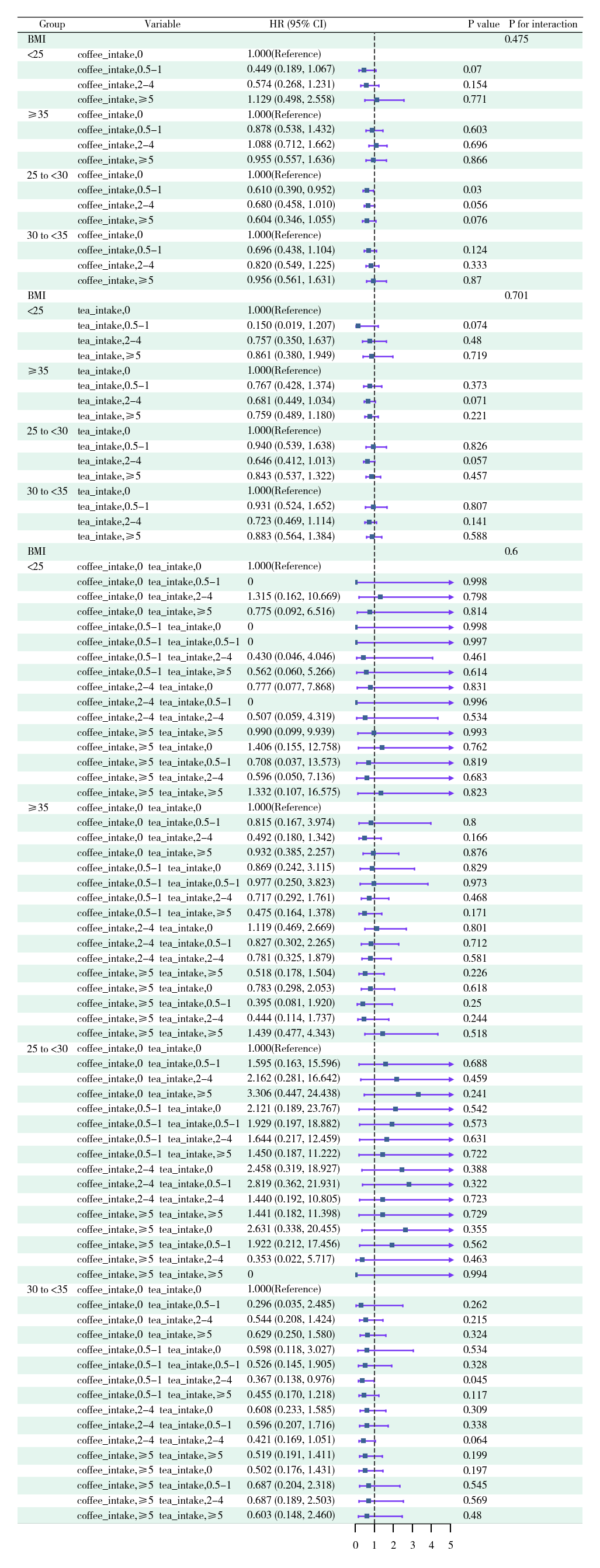
**

**Figure S5 .** Association of coffee and tea with stroke in the UK Biobank cohort by BMI. Multivariable model is adjusted for age, sex, ethnicity, qualification, income, Physical activity, smoking status, alcohol status, diet pattern , HDL, LDL, hypertension and we adjusted for coffee in tea analysis or for tea in coffee analysis. Abbreviations: CI, confidence interval; HR, hazard ratio; UK Biobank, United Kingdom Biobank; BMI, body mass index; HDL, high-density lipoprotein; LDL, low-density lipoprotein.

**
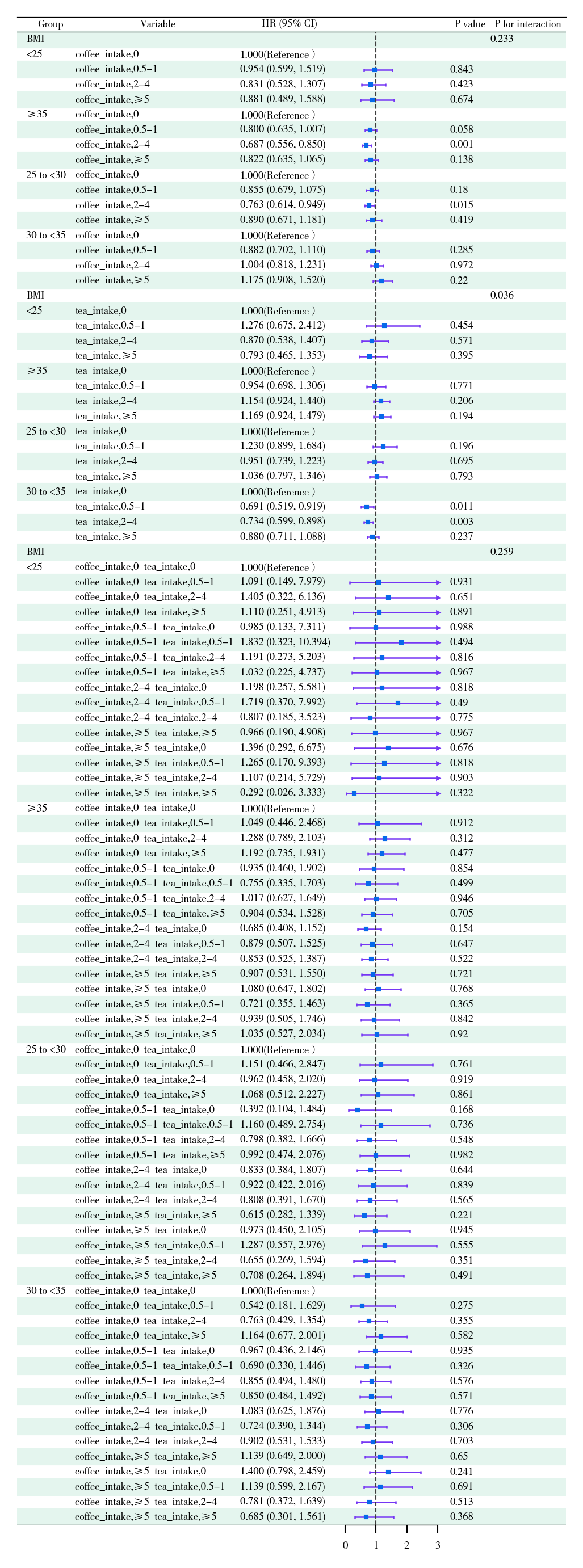
**

**Figure S6 .** Association of coffee and tea with AP in the UK Biobank cohort by BMI. Multivariable model is adjusted for age, sex, ethnicity, qualification, income, Physical activity, smoking status, alcohol status, diet pattern , HDL, LDL, hypertension and we adjusted for coffee in tea analysis or for tea in coffee analysis. Abbreviations: CI, confidence interval; HR, hazard ratio; UK Biobank, United Kingdom Biobank; BMI, body mass index; HDL, high-density lipoprotein; LDL, low-density lipoprotein.

**
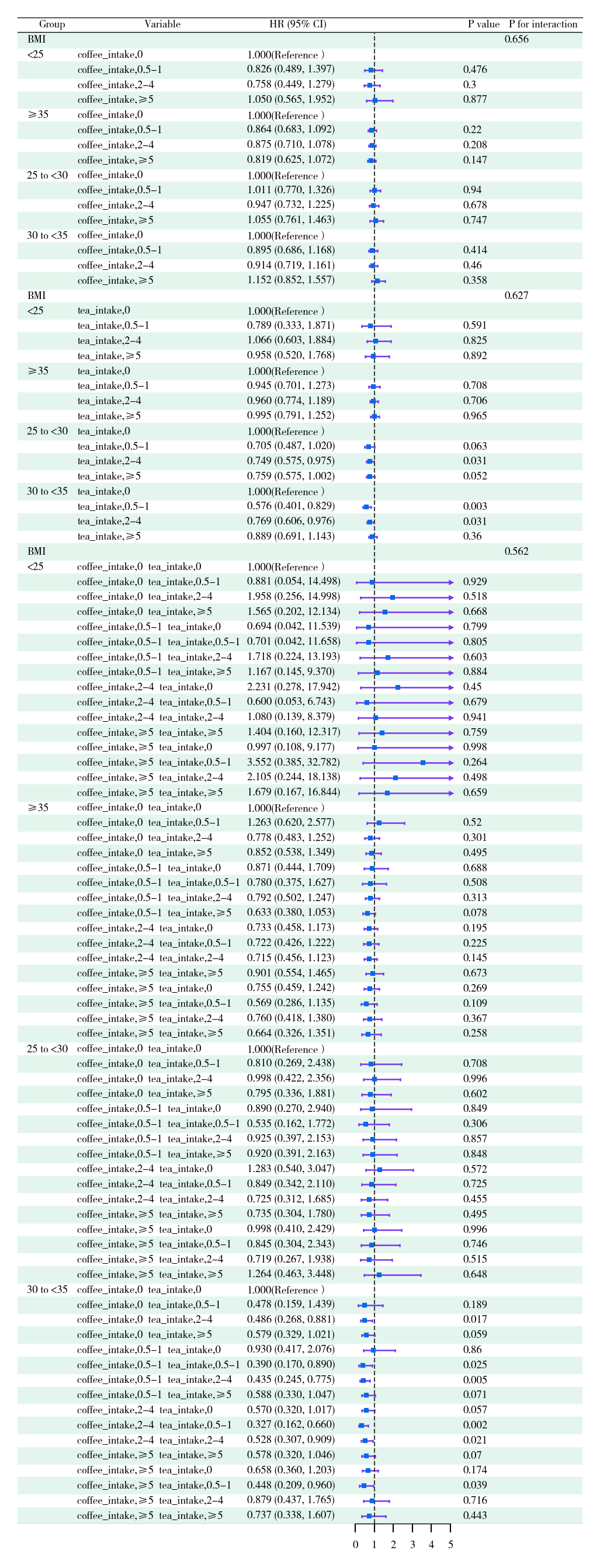
**

**Figure S7 .** Association of coffee and tea with HF in the UK Biobank cohort by BMI. Multivariable model is adjusted for age, sex, ethnicity, qualification, income, Physical activity, smoking status, alcohol status, diet pattern , HDL, LDL, hypertension and we adjusted for coffee in tea analysis or for tea in coffee analysis. Abbreviations: CI, confidence interval; HR, hazard ratio; UK Biobank, United Kingdom Biobank; BMI, body mass index; HDL, high-density lipoprotein; LDL, low-density lipoprotein.
